# Targeting FEN1 disrupts EIF4A3-mediated MUC1-C autoregulation: a novel strategy to suppress lung adenocarcinoma progression

**DOI:** 10.1038/s41419-026-09260-5

**Published:** 2026-09-05

**Authors:** Min Wu, Benmeng Wu, Xuebing Yan, Zirui Wang, Chunyan Mei, Xiaoshan Huang, Guorui Li, Ji Pang, Lin Yu, Jingjing Liu

**Affiliations:** 1 Department of Oncology, The Affiliated Hospital of Yangzhou University, Yangzhou University, Yangzhou, PR China; 2 The First School of Clinical Medicine, Faculty of Medicine, Yangzhou University, Yangzhou, PR China; 3 Department of Clinical Laboratory, The Second Affiliated Hospital of Xuzhou Medical University, Xuzhou, PR China; 4 Department of Pathology, The Affiliated People’s Hospital of Jiangsu University, Zhenjiang, PR China; 5 Department of Clinical Laboratory, The Affiliated People’s Hospital of Jiangsu University, Zhenjiang, PR China; 6 School of Basic Medical Sciences & School of Public Health, Faculty of Medicine, Yangzhou University, Yangzhou, PR China; 7 School of Traditional Chinese Medicine, Faculty of Medicine, Yangzhou University, Yangzhou, PR China

**Keywords:** Non-small-cell lung cancer, Cancer microenvironment

## Abstract

Flap endonuclease 1 (FEN1), a key enzyme in DNA metabolism, exhibits oncogenic properties in various cancers. However, its functional role and underlying mechanisms in lung adenocarcinoma (LUAD) progression and tumor immunity remain poorly defined. Here, we demonstrate that FEN1 is significantly upregulated in LUAD tissues and serves as an independent prognostic factor for poor survival. Genetic depletion of FEN1 suppressed LUAD cell proliferation by inducing G0/G1 cell cycle arrest and cellular senescence, while significantly impairing migration and invasion capabilities. Mechanistically, integrated proteomic and molecular analyses revealed that FEN1 knockdown disrupts the carboxyl-terminal subunit of mucin 1 (MUC1-C)/PI3K/AKT autoregulatory loop, a critical driver of oncogenic signaling. Furthermore, FEN1 downregulation inhibited the MUC1-C/p65/ programmed death-ligand 1 (PD-L1) signaling axis, thereby alleviating tumor-mediated immunosuppression. This was evidenced by enhanced antitumor immunity, characterized by the local expansion of CD4^+^ T cells and restored effector function of CD8^+^ T cells, both in vitro and in vivo. Mechanistically, independent of its nuclease activity, FEN1 interacts with the N-terminus of eukaryotic translation initiation factor 4A3 (EIF4A3) to facilitate its binding to MUC1-C mRNA. Specifically, FEN1 is required for EIF4A3-mediated maintenance of both the transcript stability and translation efficiency of MUC1-C. Clinically, FEN1, MUC1-C, and PD-L1 are coordinately upregulated in LUAD tissues, and their co-expression predicts poor patient survival irrespective of EGFR mutation status. Our findings establish the FEN1/EIF4A3/MUC1-C axis as a novel mechanism driving LUAD progression by concurrently regulating intrinsic malignancy and extrinsic immune evasion, presenting FEN1 targeting as a promising dual-hit therapeutic strategy.

## Introduction

Lung cancer is a malignant tumor with the highest morbidity and mortality worldwide, of which lung adenocarcinoma (LUAD) is the most common pathological type, accounting for about 40% of lung cancer [1]. The five-year survival rate for this malignancy remains low, typically ranging from 5% to 20% [2]. Therapeutic options for LUAD include surgery, radiation, chemotherapy, targeted therapy, and immunotherapy or a combination of these treatments [3]. Recent advancements in targeted therapies against several oncogenic drivers (e.g., epidermal growth factor receptor mutation and anaplastic lymphoma kinase rearrangement), and immunotherapies targeting immune checkpoints (programmed death-1 (PD-1) or programmed death ligand 1 (PD-L1)) have achieved remarkable success [4]. However, a substantial subset of LUAD patients lacks actionable targets, either due to the absence of known driver mutations or the presence of historically “undruggable” oncogenes such as *MYC* [4]. Furthermore, intrinsic and acquired resistance to targeted therapies is usually observed in lung cancer patients [5]. Consequently, there is an urgent imperative to identify novel molecular targets and devise potent therapeutic strategies for addressing this unmet clinical need.

Flap endonuclease 1 (FEN1) is a structure-specific nuclease that mediates multiple DNA metabolic pathways, including Okazaki fragment maturation, long-patch base excision repair, telomere stability maintenance, and apoptotic DNA fragment processing [6]. On one hand, FEN1 deficiency drives genomic instability and heightens tumor susceptibility [7]. On the other hand, FEN1 acts as a proto-oncogene to facilitate tumor initiation and progression. In hepatocellular carcinoma (HCC), FEN1 facilitated the proliferation, migration, invasion, and therapy resistance of cancer cells by enhancing ubiquitin-specific peptidase 7 / mouse double minute 2 homolog -mediated P53 inactivation [8]. In cholangiocarcinoma, FEN1 mediated the epithelial-mesenchymal transition process of cancer cells via the Wnt/β-catenin signaling pathway [9]. Therefore, targeting FEN1 has been verified as an effective strategy in cancer treatment [10]. In breast cancer, the inhibition of FEN1 leads to impaired DNA repair and repressed cancer progression [11]. Additionally, knockdown of FEN1 facilitates breast cancer cell senescence through pre-B-cell leukemia transcription factor 1(PBX1) down-regulation mediating an increase in intracellular ROS levels [12]. Beyond these non-immunological functions, the association between FEN1 and tumor immunity has been the subject of emerging investigation. In oral squamous cell carcinoma (OSCC), the acquisition of key immunosuppressive phenotypes exhibits a positive correlation with FEN1 expression levels [13]. In head and neck squamous cell carcinoma (HNSCC) cells, the combined strategy of FEN1 downregulation and PD-1 blockade exerts a synergistic effect in enhancing the antitumor activity of CD8 ^+ ^T cells in vitro [14]. Nevertheless, the mechanisms by which FEN1 regulates the tumor immune microenvironment remain unclarified in the aforementioned studies. Notably, research on FEN1 in LUAD remains limited; specifically, whether FEN1 regulates the malignant phenotype and immune function of LUAD cells has not yet been elucidated.

Herein, we identified that FEN1 is upregulated in LUAD tissues and indicates poor prognosis. FEN1 downregulation not only suppressed the malignant phenotype of LUAD cells, but also enhanced anti-tumor immunity. Notably, we found Mucin 1 (MUC1) to be a potential downstream target of FEN1. Collectively, these findings unravel the dual role of FEN1 in regulating LUAD cell malignancy and tumor immunity, thereby offering a theoretical basis for advancing FEN1-targeted therapies in LUAD.

## Materials and methods

### Bioinformatic analysis

LUAD RNA-seq data was downloaded from The Cancer Genome Atlas (TCGA) database (https://www.cancer.gov/tcga), generated on the Illumina HiSeq platform. The dataset included 518 tumor samples and 59 adjacent normal samples. Gene expression profiles were derived from raw sequencing data and normalized to transcripts per million reads (TPM) using the R package “tximport”. The 518 tumor samples and 59 adjacent normal samples were used for analyzing FEN1 expression levels, while the correlation between FEN1 expression (high vs. low) and clinical data of 518 LUAD patients was evaluated. Prognostic analysis was conducted on 516 tumor samples with complete survival information. R packages “survival” and “survminer” were used to perform Kaplan-Meier survival analyses, as well as univariate and multivariate Cox regression analyses. Nomograms were constructed using the R packages “survival” and “rms”. The correlations between FEN1 expression and immune regulation-related molecules were analyzed using the Tumor Immune Estimation Resource, version 2 (TIMER2.0) database (http://timer.cistrome.org/), with Spearman’s correlation coefficients calculated.

### LUAD specimens

This study was approved by the Ethical Committee of the Affiliated Hospital of Yangzhou University, and written informed consent was obtained from all participating patients. A total of 49 pairs of LUAD tissues and adjacent normal tissues were collected from patients with pathologically confirmed LUAD at the Affiliated Hospital of Yangzhou University (Yangzhou, Jiangsu, China) between March 2024 and July 2024. Inclusion criteria:(a) LUAD diagnosed by two independent pathologists, with TNM stage I - IV (based on the 8th edition of AJCC staging system); (b) Complete clinicopathological data and MRI information available;(c) No history of antitumor therapy (including surgery, chemotherapy, radiotherapy, or targeted therapy) prior to tissue collection. Exclusion criteria:(a) Complicated with other malignant tumors;(b) Incomplete clinical data.

### Clinical database analysis

Clinical data analysis was performed using publicly available datasets. Overall survival curves based on the co-expression of FEN1, MUC1-C, and PD-L1 (or FEN1 and MUC1-C) were generated using the Kaplan-Meier Plotter database. Patients were stratified into high and low expression groups based on the median of the mean expression levels of the selected multigene panels. For correlation analysis, gene expression and somatic mutation data from the TCGA-LUAD cohort were downloaded. Pearson correlation coefficients were calculated to assess the relationship between FEN1 and PD-L1 mRNA levels in patients stratified by EGFR mutation status.

### Cell culture and stable cell lines

Laboratory-preserved human LUAD cells (A549, PC9), Lewis Lung Carcinoma (LLC) cells, and HEK293T cells (Pricella, China) were cultured in RPMI 1640 or DMEM, supplemented with 10% fetal bovine serum (FBS) and 1% penicillin-streptomycin, at 37 °C in a humidified incubator with 5% CO₂. To establish stable FEN1-knockdown cell lines, A549, PC9, and LLC cells were transfected with lentiviral vectors carrying FEN1 shRNA or a scrambled shRNA control vector, which were synthesized by Hanbio (Shanghai, China). Subsequently, transfected cells were subjected to a two-week puromycin selection, and the knockdown efficiency was confirmed by Western blot.

### Western blot analysis

Cells were harvested and lysed in radioimmunoprecipitation assay (RIPA) buffer (Solarbio, Beijing, China) supplemented with a protease inhibitor cocktail (Roche, Switzerland). Protein concentrations were determined using a bicinchoninic acid (BCA) assay kit (Yeasen, Shanghai, China). Protein samples (20 µg per lane) was separated by 8–12% SDS-PAGE and transferred onto polyvinylidene fluoride (PVDF) membranes (Sigma, Germany). The membranes were blocked with 5% skim milk and subsequently incubated with the appropriate primary antibodies (listed in supplementary Table 1), followed by incubation with corresponding secondary antibodies (listed in Supplementary Table 1). Protein bands were visualized using an Electrophoresis Gel Imaging Analysis System (Tanon, China), and band intensities were quantified with ImageJ software. Relative protein expression levels were normalized to GAPDH as the loading control.

### MTT assay

Cell suspensions were prepared at 1 × 10⁵ cells/mL and plated in 96-well plates (100 μL per well), followed by incubation at 37 °C under 5% CO₂ for the designated times. After replacing the culture medium with 100 μL basal medium, 10 μL of MTT solution was added to each well, and the plates were incubated in the dark for 2 h. The resulting formazan crystals were dissolved by adding 150 μL of DMSO per well after removing the medium. The absorbance was then read at 492 nm on a microplate reader following thorough mixing.

### Cell cycle distribution

After trypsinization and washing, cells (4 × 10⁵ per well) were fixed in 70% ice-cold ethanol at 4 °C overnight. Subsequently, cells were washed twice with ice-cold PBS and resuspended in RNase A at 37 °C for 30 min. Then, cells were stained with propidium iodide (PI) in the dark at 4 °C for 30 min. Cell cycle distribution was detected using a BD FACSVerse™ Cell Analyzer (BD Biosciences, USA), and quantified with FlowJo software.

### Annexin V-APC/ PI assay

After washing twice with ice-cold PBS, cells (4 × 10⁵ per tube) were resuspended in 100 μL of 1× Annexin V Binding Buffer and stained with 2.5 μL of Annexin V-APC and 2.5 μL of PI. Following gentle vortexing, the mixture was incubated at room temperature in the dark for 15 min. Subsequently, 400 μL of 1× Annexin V Binding Buffer was added to each tube and mixed thoroughly. Apoptosis analysis was then performed using a BD FACSVerse™ Cell Analyzer (BD Biosciences, USA) and quantified with FlowJo software.

### SA-β-gal staining assay

SA-β-Gal Staining Kit (Solarbio, Beijing, China) was employed to detect cell senescence. Cells (8 × 10⁵ per well) were rinsed with PBS and fixed with 1 mL of β-Gal staining fixative solution for 15 min at room temperature. Then, cells were washed three times with PBS for 3 min each and incubated with 1 mL of β-Gal staining solution overnight at 37 °C. Stained cells stored in PBS were visualized under a light microscope, and the senescence rate was quantified with ImageJ software across three randomly chosen fields.

### Transwell assay

Cell migration assay was performed using 24-well Transwell chambers with 8 μm pores (Corning, USA). Briefly, the lower chamber was filled with 500 μL of medium supplemented with 10% FBS, while 300 μL of serum-free cell suspension (8 × 10⁵ cells/mL) was added to the upper chamber. After 18 h-incubating, the cells were washed with PBS, fixed with 4% paraformaldehyde for 15 min, and stained with crystal violet for 20 min at room temperature. Following PBS washing and air-drying, migrated cells on the lower chamber membrane were counted in three random fields.

For the invasion assay, the upper membrane of the chamber was pre-coated with Matrigel matrix (Corning, USA), while the remaining experimental procedures were otherwise identical to the migration assay.

### Proteomic analysis

Proteomic analysis was performed on 5 × 10⁶ A549 cells (sh-NC and sh-FEN1#2 groups) with support from Beijing Biomarker Corporation (Beijing, China). The procedures included protein extraction, quantification, tryptic digestion, and peptide quantification, followed by liquid chromatography-tandem mass spectrometry (LC-MS/MS). Raw mass spectrometry data were processed using Proteome Discoverer (version 2.4 SP1). All analyses were conducted with three independent biological replicates per group.

### Immunoprecipitation (IP) -MS assay for identification of FEN1-interacting proteins

A549 cells (1 × 10⁷) were lysed on ice using RIPA buffer supplemented with 1% PMSF. The lysate was divided into equal aliquots for incubation with control IgG or anti-FEN1 antibody (listed in supplementary Table 1) overnight at 4 °C on a rotator. Immunocomplexes were captured by subsequent incubation with Protein A/G beads (Beyotime, China) for 3 h. Beads were then collected by centrifugation, washed thoroughly three times with PBS, and resuspended in 5× SDS loading buffer (NCM Biotech, China). After boiling at 95 °C for 5 min, proteins were separated by SDS-PAGE and visualized with Coomassie Blue staining. Excised gel bands were submitted to Genechem (Shanghai, China) for protein identification by LC-MS/MS.

### Small interfering RNA (siRNA) and plasmid transfection

siRNA targeting FEN1 and plasmids were synthesized by Gene Create (Wuhan, China). The sense strand sequence for si-FEN1 was 5’-GACUGCCAGUGAAGCCAAA-3’. Cells at 80% or 60% confluence were transfected with siRNA or plasmid using Lipofectamine 3000 (Thermo Fisher, USA) according to the manufacturer’s protocol. After 24–48 h incubation, cells were harvested for subsequent experiments.

### Surface sensing of translation (SUnSET) assay for global translation

Global protein synthesis was evaluated using SUnSET assay. Briefly, cells cultured in 6-well plates were treated with 5 μg/mL puromycin (Beyotime, China) for 30 min at 37 °C before harvesting. Cells were then washed with cold PBS and lysed in RIPA buffer. The amount of newly synthesized proteins was determined by Western blotting using an anti-puromycin antibody (listed in supplementary Table 1).

### Nuclear and cytoplasmic fractionation

Cells were fractionated into nuclear and cytoplasmic components using a Nuclear and Cytoplasmic Extraction Kit (Beyotime, China) according to the manufacturer’s instructions. The purity of the fractions was verified by Western blotting using antibodies against Lamin A/C (nuclear marker, listed in supplementary Table 1) and GAPDH (cytoplasmic marker). Co-IP assays were then performed separately on the isolated nuclear and cytoplasmic fractions as described above.

### Co-IP assay

Exogenous protein interaction: HEK293T cells, A549 and PC9 cells were seeded in culture dishes and grown to 60% confluence. Cells were co-transfected with Flag-tagged FEN1 plasmids (full-length or truncation mutants) and His-tagged EIF4A3 plasmids (full-length or truncation mutants) at a mass ratio of 1:1. At 48 h post-transfection, cells were lysed on ice using RIPA buffer supplemented with 1% PMSF, as described in the “Immunoprecipitation (IP) - MS assay for identification of FEN1-interacting proteins” section. Equal amounts of cell lysates were incubated with anti-Flag antibody or anti-His antibody (listed in supplementary Table 1) overnight at 4 °C on a rotator (normal IgG served as negative control), followed by incubation with Protein A/G beads (Beyotime, China) for 3 h to capture immunocomplexes.

Endogenous protein interaction: A549 and PC9 cells were cultured to 90% confluence and were lysed on ice using RIPA buffer supplemented with 1% PMSF, following the same lysis protocol as the exogenous Co-IP assay. Equal amounts of cell lysates were incubated with anti-FEN1 antibody or anti-EIF4A3 antibody (listed in supplementary Table 1) overnight at 4 °C on a rotator (normal IgG served as negative control). Immunocomplexes were captured by incubation with Protein A/G beads (Beyotime, China) for 3 h.

Protein detection: For both exogenous and endogenous Co-IP assays, beads were collected by centrifugation, washed three times with PBS, and resuspended in 5× SDS loading buffer. After boiling at 95 °C for 5 min, the co-precipitated proteins were separated by SDS-PAGE and detected by Western blot using corresponding primary antibodies and secondary antibodies (listed in supplementary Table 1).

To eliminate nucleic acid-mediated protein interactions, cell lysates were subjected to nuclease treatment prior to immunoprecipitation. Briefly, the clarified lysates were incubated with 250 U/mL Benzonase nuclease (Sigma-Aldrich, USA) supplemented with 2 mM MgCl_2_ for 30 min at 4 °C to digest DNA and RNA. Following digestion, the reciprocal Co-IP steps were performed using specific antibodies and Protein A/G magnetic beads as described above.

### Plasmids and site-directed mutagenesis

The coding sequence of FEN1 was cloned into the pCDNA3.1-Flag vector to generate N-terminally Flag-tagged FEN1. The nuclease-dead FEN1 mutant (D181A) was generated by site-directed mutagenesis using the QuikChange Lightning Kit (Agilent Technologies, USA) according to the manufacturer’s instructions. The aspartic acid at position 181 was replaced with alanine. The coding sequence of EIF4A3 was cloned into the pCDNA3.1-His vector to generate N-terminally His-tagged EIF4A3. All constructs were verified by Sanger sequencing. Wild-type and mutant FEN1 plasmids, as well as His-EIF4A3, were used for transient transfection.

### Immunofluorescence

Cells (4 × 10⁵/well) grown on coverslips were washed with PBS, fixed with 4% paraformaldehyde for 20 min, and permeabilized with 0.5% Triton X-100 for 10 min at room temperature. After blocking with 2% bovine serum albumin for 1 h, cells were incubated overnight at 4 °C with primary antibodies (listed in supplementary Table 1) diluted in 2% BSA, followed by incubation with corresponding secondary antibodies (listed in supplementary Table 1) for 1 h at room temperature in the dark. Nuclei were counterstained with DAPI (Santa Cruz, CA, USA). Images were acquired using either a fluorescence microscope or a laser scanning confocal microscope (ZEISS, Germany) equipped with a 63×/1.40 oil objective and 340 nm, 488 nm, and 594 nm laser lines. Image analysis was performed with ImageJ software.

For the tissue sections, following deparaffinization, rehydration, and antigen retrieval, sections were blocked with 5% BSA at 37 °C for 30 min. The subsequent steps were consistent with those described for cell immunofluorescence staining.

### Actinomycin D (Act D) chase assay

To measure mRNA stability, cells were treated with 12.5 ng/mL (A549) or 0.4 ng/mL (PC9) Actinomycin D (Beyotime, China) to inhibit de novo transcription. Total RNA was extracted at designated time points (0, 3, 6, 9 and 12 h) post-treatment using TRIzol reagent. MUC1-C mRNA levels were quantified via RT-qPCR and normalized to GAPDH. The relative mRNA remaining at each time point was calculated compared to the 0-hour baseline.

### RNA-binding protein immunoprecipitation-quantitative real-time PCR (RIP-qPCR) assay

The RIP assays were conducted using the RNA-Binding Protein Immunoprecipitation Kit (Genecreate, China). LUAD cells (2 × 10⁷) were lysed in 400 µL of RIP lysis buffer containing protease and RNase inhibitors, followed by centrifugation at 12,000 × *g* for 15 min at 4 °C. The cell lysates were then incubated with either IgG or anti-EIF4A3 antibody-conjugated magnetic beads (Beyotime, China) and rotated at 4 °C overnight. The beads were washed five times with lysis buffer, and RNA was purified using chloroform extraction. Finally, the enrichment of MUC1 mRNA was quantified by qPCR.

Translation efficiency was evaluated using a ribosome RNA immunoprecipitation (Ribo-RIP) assay. Cells were treated with 100 μg/mL cycloheximide (Beyotime, China) for 15 min to stall translating ribosomes prior to harvest. Cell lysates were prepared and incubated with an anti-RPL10a antibody (listed in supplementary Table 1) or an IgG control at 4 °C overnight, followed by incubation with Protein A/G magnetic beads. After washing the beads, the co-immunoprecipitated RNA was extracted. The enrichment of MUC1-C mRNA in the actively translating ribosome complex was quantified using RT-qPCR and normalized to the input RNA.

### In vivo tumor model experiments

Four-week-old female BALB/c nude mice and C57BL/6 mice were obtained from the Animal Research Center of Yangzhou University (Yangzhou, China) and acclimatized for one week prior to experimentation. For the establishment of xenograft model, 5 × 10^6^ of FEN1 knockdown (sh-FEN1#2) or control (sh-NC) A549 cells suspended in 100 μL PBS were subcutaneously inoculated into the right flanks of the nude mice (*n *= 5). Tumor growth was monitored every five days by measuring the longest (L) and shortest (W) dimensions with a vernier caliper, and volume was calculated using the formula V = (L × W²)/2. All mice were euthanized 30 days post-inoculation, and tumors were harvested for further research.

For the construct of Lewis model, 2 × 10^5^ of LLC cells (sh-FEN1#2 or sh-NC) were resuspended in 100 μL PBS and injected into the flanks of C57BL/6 mice (*n* = 4). Tumor growth was monitored every three days. All mice were euthanized 18 days post-inoculation, and tumors were harvested for further research.

### Human CD3^+^ T cell isolation

Human peripheral blood samples were obtained from healthy donors with written informed consent. Peripheral blood mononuclear cells (PBMCs) were isolated from whole blood samples obtained from healthy human donors using Human Peripheral Blood Lymphocyte Isolation Solution (Solarbio, Beijing, China) according to the manufacturer’s protocol. 1 × 10⁷ cells/mL PBMCs were resuspended in 80 μL PBS, followed by incubating with 20 μL CD3 MicroBeads (Miltenyi Biotec, Germany) for 15 min at 4 °C. The cell-bead mixture was then diluted with 2 mL PBS and centrifuged. After discarding the supernatant, the mixture was resuspended in 500 μL PBS, followed by loaded onto an LS column pre-rinsed with PBS and positioned on a magnetic stand. Following three washes with PBS to remove unlabeled cells, the column was transferred to a clean centrifuge tube. The magnetically labeled CD3⁺ T cells were eluted three times by flushing with 1 mL PBS under plunger pressure. The isolated CD3⁺ T cells were resuspended in RPMI 1640 at 1 × 10⁶ cells/mL, and then transferred to 6-well cell culture plates which containing 2 μg/mL anti-CD3 and 2 μg/mL anti-CD28 antibodies for 72 h-incubation in a 37 °C, 5% CO2 incubator prior to subsequent experiments.

### LUAD cell and T cell co-culture assay

LUAD cells (A549 and PC9) were seeded in 96-well plates at a density of 1 × 10⁵ cells/mL and cultured for 24 h. Activated human CD3⁺ T cells (isolated and activated as described in the “Human CD3⁺ T cell isolation” section) were then added to the adherent LUAD cells for co-culture at a T cell: LUAD cell ratio of 10:1. The co-culture system was maintained in RPMI 1640 medium supplemented with 10% FBS and 1% penicillin-streptomycin at 37 °C in a 5% CO₂ incubator for 48 h. After co-culture, non-adherent T cells were collected for flow cytometry analysis. Adherent LUAD cells were washed three times with PBS, and their viability was evaluated using the MTT assay.

### Flow cytometry analysis of immune cell phenotypes and cytokines

For co-culture assay, collected T cells were washed twice with PBS and resuspended in 200 μL PBS containing antibodies including PE anti-human CD3, FITC anti-human CD4 and APC anti-human CD8 (listed in Supplementary Table 1) for 30 min on ice. For intracellular staining, cells were fixed and permeabilized using the eBioscience^TM^ FOXP3/Transcription Factor Staining Buffer Set (Thermo Fisher Scientific, USA), followed by incubation with PE/Cyanine7 anti-human/mouse Granzyme B, Brilliant Violet 421™ anti-human TNF-α and RB705 anti-human IFN-γ (listed in Supplementary Table 1) for 30 min on ice. Cells were then subjected to flow cytometry analysis by CytoFLEX flow cytometer (Beckman Coulter, USA). Data were analyzed with FlowJo software.

For mouse samples, fresh tumour tissue was excised and digested with hyaluronidase, collagenase IV and DNase I (Solarbio, Beijing, China) for 25 min at 37 °C with 200 rpm shaking, followed by filtration through 40 μm cell strainers to obtain a single-cell suspension. The samples were then blocked with 2% BSA, and dead cells were excluded using the LIVE/DEAD™ Fixable Near IR (780) Viability Kit (Thermo Fisher, USA). For immunophenotyping, cells were stained with surface markers including FITC anti-mouse CD45, BV510 anti-mouse CD3e, APC anti-mouse CD4, and PerCP-Cy™5.5 anti-mouse CD8a (listed in Supplementary Table 1). After fixation and permeabilization using a eBioscience^TM^ FOXP3/Transcription Factor Staining Buffer Set, cells were incubated with the following antibodies: PE/Cyanine7 anti-human/mouse Granzyme B, PE anti-mouse TNF-α, PE anti-mouse IFN-γ (listed in Supplementary Table 1). Stained cells were analyzed by CytoFLEX flow cytometer, and data were quantified using FlowJo software.

### T cell chemotaxis, proliferation and exhaustion assays

For the chemotaxis assay, tumor cells were cultured in six-well plates. Upon adherence, the medium was replaced with serum-free medium and cultured for an additional 24 h. The supernatants were collected and filtered through a 0.22 μm filter to prepare the conditioned media (CM). Activated T cells were labeled with Calcein-AM and suspended in serum-free medium. Then, $2\times {10}^{5}$ labeled T cells were seeded into the upper chambers of 24-well Transwell inserts (5 μm pore size). The lower chambers were filled with the corresponding CM. After 4 h of incubation, the inserts were removed, and the migrated cells in the lower chamber were photographed and quantified using a fluorescence microscope. The migration index was expressed as the cell number relative to the control group.

For the proliferation assay, T cells were harvested from the tumor cell co-culture system after 48 h and pulsed with 10 μM EdU for 2 h in fresh medium. Following incubation, cells were fixed with 4% paraformaldehyde and subjected to the Click-it reaction according to the manufacturer’s protocol (Beyotime, China). To specifically identify the CD4^+^ subset, cells were subsequently incubated with a fluorescently-conjugated anti-CD4 antibody (1:100) in PBS containing 2.5% BSA for 30 min at 4 °C. The percentage of EDU-positive cells within the CD4^+^ gated population was analyzed using a flow cytometer.

For T cell exhaustion analysis, total T cells harvested from the co-culture system were washed and stained with fluorescently conjugated anti-CD8, alongside anti-PD-1 (listed in Supplementary Table 1) and anti-TIGIT (listed in Supplementary Table 1) antibodies. Data were acquired using a flow cytometer and analyzed with FlowJo software. The expression levels of PD-1 and TIGIT were evaluated by specifically gating on the CD8^+^ cell population.

### Immunohistochemistry (IHC)

IHC was performed on formalin-fixed, paraffin-embedded tissue sections. Briefly, sections were baked at 60 °C for 1 h, followed by deparaffinization and rehydration. After antigen retrieval, endogenous peroxidases were blocked with 3% hydrogen peroxide, followed by blocking of nonspecific sites with 10% goat serum. Sections were then incubated overnight at 4 °C with primary antibodies (listed in Supplementary Table 1), followed by a 30 min incubation with horseradish peroxidase (HRP)-conjugated secondary antibody (listed in Supplementary Table 1) at room temperature. DAB substrate was used for the chromogenic reaction, and nuclei were counterstained with hematoxylin. Staining results were evaluated based on the staining intensity score [15].

### Statistical analysis

Results were analyzed using the GraphPad Prism 9 software. All experiments were performed with at least three independent replicates, and data are presented as mean ± standard deviation (SD). Differences between two groups were evaluated by chi-square test or Student’s t-test, while multiple group comparisons were analyzed using one-way analysis of variance (ANOVA). Statistical significance was defined as *P* < 0.05.

## Results

### FEN1 is upregulated in LUAD tissues and indicates poor prognosis

**Fig. 1:**
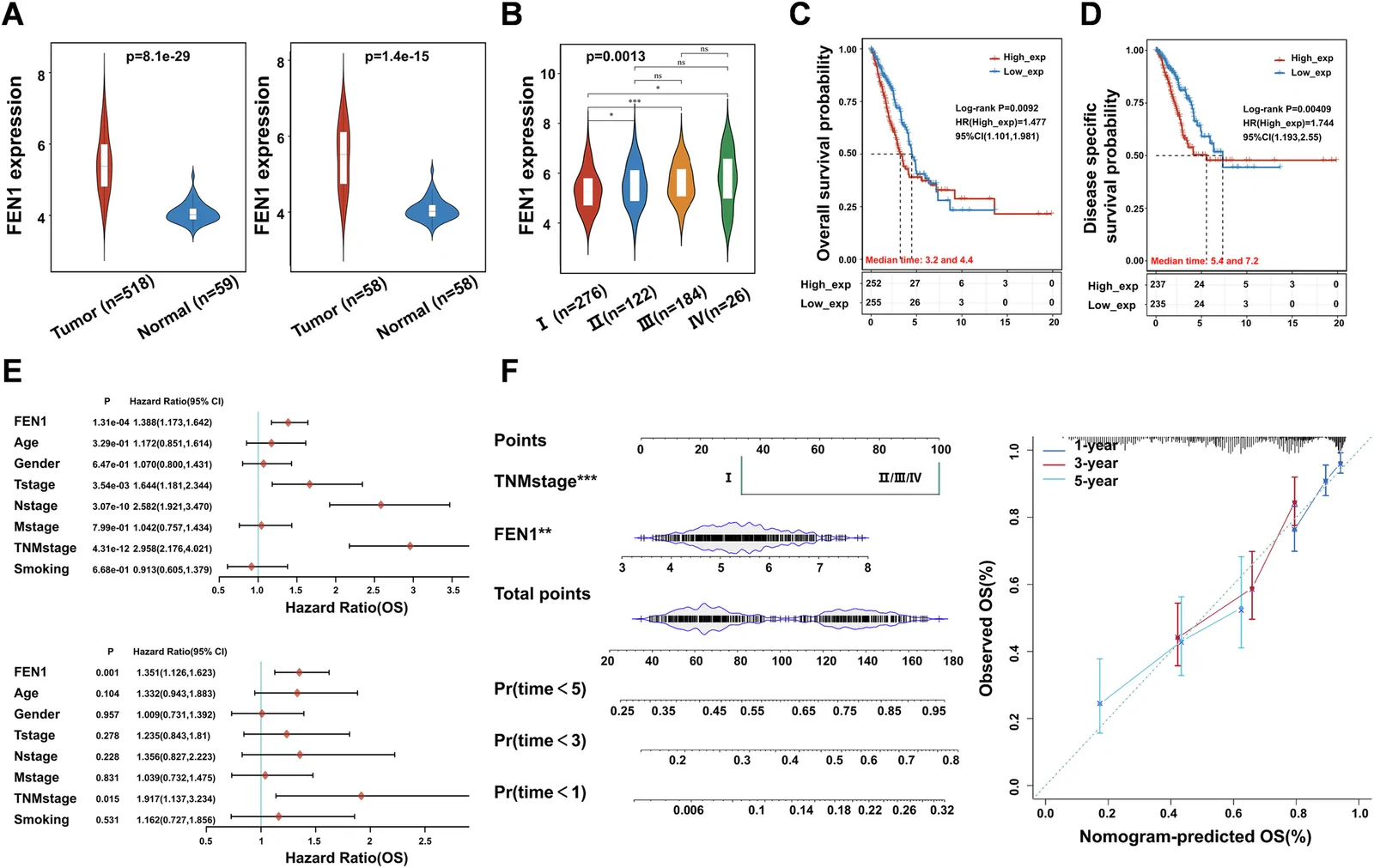
FEN1 is upregulated in LUAD tissues and indicates poor prognosis. **A** FEN1 expression levels in LUAD and adjacent normal tissues (left); FEN1 expression levels in LUAD and paired normal tissues (right). **B** FEN1 expression levels in LUAD grouped by TNM stages. **C** Kaplan–Meier analysis of OS for LUAD patients. **D** Kaplan–Meier analysis of DSS for LUAD patients. **E** Forest plots of the univariate and multivariate Cox regression analysis for predicting OS in LUAD patients. **F** Nomogram for predicting 1-, 3-, and 5-year OS in patients with LUAD (left), and its corresponding calibration plots (right). *P < 0.05, **P < 0.01, ***P < 0.001, ns, not significant.

TCGA-LUAD databases were used to predict FEN1 expression in 518 LUAD tissue samples and 59 normal tissue samples. The results showed that FEN1 expression levels were significantly higher in LUAD primary tumor samples than in normal tissue (Fig. 1A). Additionally, FEN1 expression was significantly upregulated in 58 LUAD samples compared to matched adjacent samples (Fig. 1A). Importantly, FEN1 expression was significantly different in different T stages, M stages, N stages (Fig. S1A–C) and clinical stages (Fig. 1B).

Based on the expression characteristics of FEN1, we further evaluated its clinical significance in LUAD. The characteristics of 518 LUAD patients, including clinical and gene expression data, were collected from the TCGA database. The patients were divided into high and low FEN1 expression groups based on the mean value of FEN1 expression. In the TCGA cohort, FEN1 expression was significantly associated with age and TNM stage but not with gender, M stage, and smoking history (Fig. S1D–H). In detail, patients with high FEN1 expression were younger, and patients with low FEN1 expression accounted for a higher proportion of stage Ⅰ.

Survival analysis demonstrated that high FEN1 expression was correlated with poor overall survival (OS) and poor disease-specific survival (DSS) (Fig. 1C, D). Univariate Cox regression analysis indicated that FEN1 expression, T stage, N stage, and TNM stage were independent factors for OS of LUAD patients (Fig.1E), while its performance for DSS was also approximately statistically significant (Fig. S1I). Further multivariate Cox regression analysis indicated that FEN1 expression and TNM stage were independent factors for OS (Fig. 1E) and DSS (Fig. S1I) of LUAD patients. Subsequently, a nomogram was constructed using TNM stage and FEN1 levels as indicators to predict 1-, 3-, and 5-year OS (Fig. 1F) or DSS (Fig. S1J) in patients with LUAD. The calibration curve presented desirable prediction of the nomograms for the 1-, 3-, and 5-year clinical outcomes (Fig. 1F, Fig. S1J). Overall, the aforementioned data demonstrated that FEN1 may serve as a valuable prognostic biomarker for LUAD patients.

### FEN1 downregulation induces senescence and impairs migration/invasion of LUAD cells

To delineate the functional role of FEN1 in LUAD, we first assessed the effects of FEN1 downregulation on the malignant phenotypes of LUAD cells. Stable FEN1-knockdown LUAD cell lines (A549 and PC9) were established via lentiviral transfection, and Western blot confirmed efficient FEN1 protein reduction (Fig. S2A). MTT assays demonstrated that FEN1 downregulation significantly suppressed the proliferation of A549 and PC9 cells relative to control cells (Fig. S2B). Flow cytometric analysis further revealed that FEN1 knockdown induced cell cycle arrest at the G0/G1 phase (Fig. S2C). Given the observed proliferation inhibition and cell cycle arrest, we next evaluated cell senescence and apoptosis. As shown in Fig. S2D–E, FEN1 downregulation significantly promoted LUAD cell senescence but had no detectable impact on apoptosis. We further investigated the role of FEN1 in LUAD cell migration and invasion using Transwell assays. Relative to controls, FEN1 downregulation significantly reduced the number of migrated cells (Fig. S2F). Additionally, FEN1 knockdown similarly decreased the number of invaded cells (Fig. S2G). These results confirm that FEN1 downregulation impairs the migratory and invasive capacities of LUAD cells. Collectively, these findings indicated that FEN1 knockdown promotes senescence and suppresses migration and invasion in LUAD cells.

### FEN1 downregulation blocks the MUC1-C/PI3K/AKT autoregulatory loop to inhibit LUAD progression

**Fig. 2:**
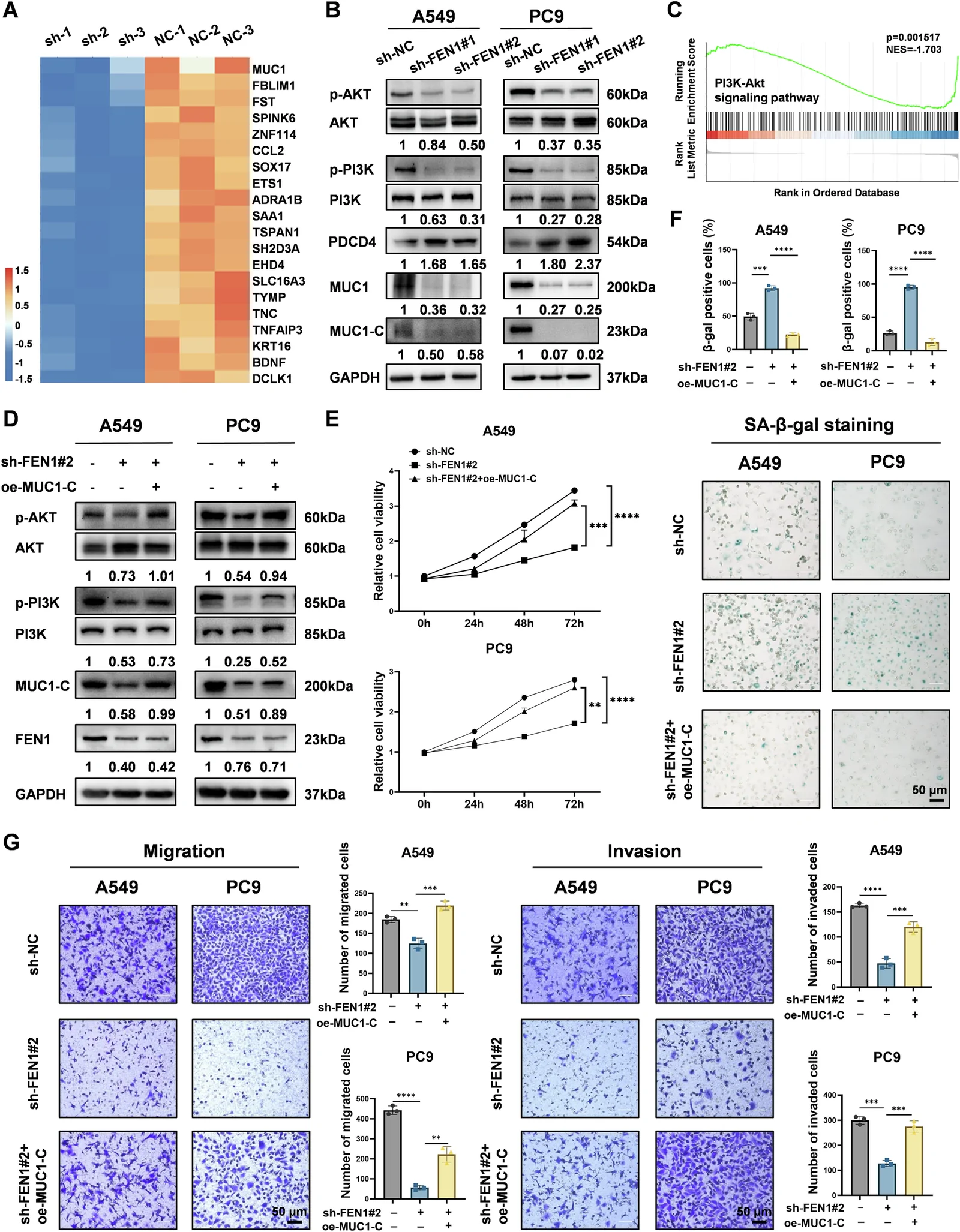
MUC1-C rescues the malignant phenotype of LUAD cells induced by FEN1 downregulation. **A** Heatmap showing downregulated proteins in FEN1knockdown cells. **B** Western blot analysis detecting the levels of p-AKT, AKT, p-PI3K, PI3K, PDCD4, MUC1, and MUC1-C after FEN1 knockdown. **C** GSEA enrichment analysis revealing the correlation between FEN1 expression and the PI3K/AKT signaling pathway. **D** Western blot analysis determining the levels of p-AKT, AKT, p-PI3K, PI3K, and MUC1-C in FEN1-downregulated cells with ectopic MUC1-C overexpression. **E** MTT assay evaluating the effect of MUC1-C overexpression on the proliferative capacity of FEN1-downregulated cells. **F** SA-β-gal staining assessing the impact of MUC1-C overexpression on the senescence of FEN1-downregulated cells. **G** Transwell assay investigating the influence of MUC1-C overexpression on the migratory and invasive abilities of FEN1-downregulated cells. Data are presented as means ± SD of three independent biological experiments. **P < 0.01, ***P < 0.001, ****P < 0.0001, ns, not significant.

To identify the mechanism by which FEN1 downregulation suppresses LUAD malignancy, we performed proteomic analysis on stable A549 cells (sh-NC vs. sh-FEN1#2). Heatmap analysis revealed that MUC1 protein levels were most significantly reduced following FEN1 knockdown (Fig. 2A). MUC1 is a transmembrane glycoprotein consisting of an extracellular N-terminal subunit (MUC1-N) and a transmembrane C-terminal subunit (MUC1-C) [16], with MUC1-C acting as the primary oncogenic moiety [17]. Notably, MUC1 is overexpressed in LUAD patients and is associated with increased tumor-infiltrating immune cells [18]. We further confirmed that FEN1 downregulation significantly decreased the protein levels of total MUC1 and its MUC1-C subunit (Fig. 2B). To confirm the positive regulation of MUC1-C by FEN1, we performed gain-of-function experiments. Wild-type LUAD cells were transfected with increasing doses of a FEN1 overexpression plasmid. Western blot analysis revealed that FEN1 overexpression increased MUC1-C protein levels in a dose-dependent manner (Fig. S3A). These results, combined with our FEN1 depletion data, demonstrate that FEN1 positively regulates MUC1-C expression in LUAD cells. Previous studies have identified a MUC1-C/ phosphoinositide 3-kinase (PI3K)/ serine/threonine kinase (AKT) autoregulatory loop that further reinforces MUC1-C overexpression in tumor cells, which sustains oncogenic signaling [19]. To investigate whether PI3K/AKT activity reciprocally regulates MUC1-C to form a feedback loop in our model, we manipulated AKT activity in LUAD cells. Treatment with the AKT inhibitor MK-2206 reduced MUC1-C protein level (Fig. S3B). Furthermore, transfection of a constitutively active AKT plasmid (ca-AKT) increased MUC1-C expression (Fig. S3C). These results suggest an ‘AKT → MUC1-C’ positive feedback mechanism in LUAD cells. The gene set enrichment analysis (GSEA) demonstrated a significant positive correlation between FEN1 expression and activation of the PI3K/AKT signaling pathway (Fig. 2C). Correspondingly, FEN1 knockdown did not alter the total protein levels of PI3K or AKT, but significantly reduced the phosphorylation levels of both PI3K and AKT (Fig. 2B). Because active AKT canonically promotes the proteasomal degradation of the translational repressor programmed cell death protein 4 (PDCD4) [19], this AKT attenuation led to a post-translational accumulation of PDCD4 (Fig. 2B), further blocking MUC1-C expression. These results indicated that FEN1 downregulation disrupts the MUC1-C/PI3K/AKT autoregulatory loop in LUAD cells.

To verify whether FEN1 regulates the malignant phenotype of LUAD cells through MUC1-C, we conducted rescue experiments by transfecting a MUC1-C overexpression plasmid into FEN1-downregulated LUAD cell lines. As shown in Fig. 2D, MUC1-C overexpression significantly restored the reduced p-PI3K and p-AKT levels caused by FEN1 knockdown. Functionally, MUC1-C overexpression reversed FEN1 knockdown-induced inhibition of cell proliferation (Fig. 2E), abrogated FEN1 knockdown-promoted cell senescence (Fig. 2F), and rescued the impaired cell migration and invasion capacities caused by FEN1 downregulation (Fig. 2G). These data confirmed that MUC1-C mediates the suppression of LUAD cell malignant phenotypes induced by FEN1 downregulation.

To extend these findings in vivo, we established a xenograft tumor model by inoculating nude mice with stable FEN1-knockdown or control A549 cells. Compared to the control group, FEN1 knockdown significantly reduced tumor volume and weight (Fig. S4A–C), demonstrating that FEN1 downregulation inhibits LUAD cell growth in vivo. Additionally, FEN1 knockdown promoted senescence (Fig. S4D) and decreased the protein expression of FEN1, MUC1-C, and the proliferation marker Ki67 in xenograft tumors (Fig. S4E). These in vivo results further validated that FEN1 downregulation reduces MUC1-C expression and suppresses LUAD tumor growth. Taken together, our findings demonstrated that FEN1 downregulation inhibits LUAD progression by blocking the MUC1-C/PI3K/AKT autoregulatory loop.

### FEN1 downregulation suppresses the MUC1-C/p65/PD-L1 signaling axis in LUAD cells and modulates antitumor immunity

**Fig. 3:**
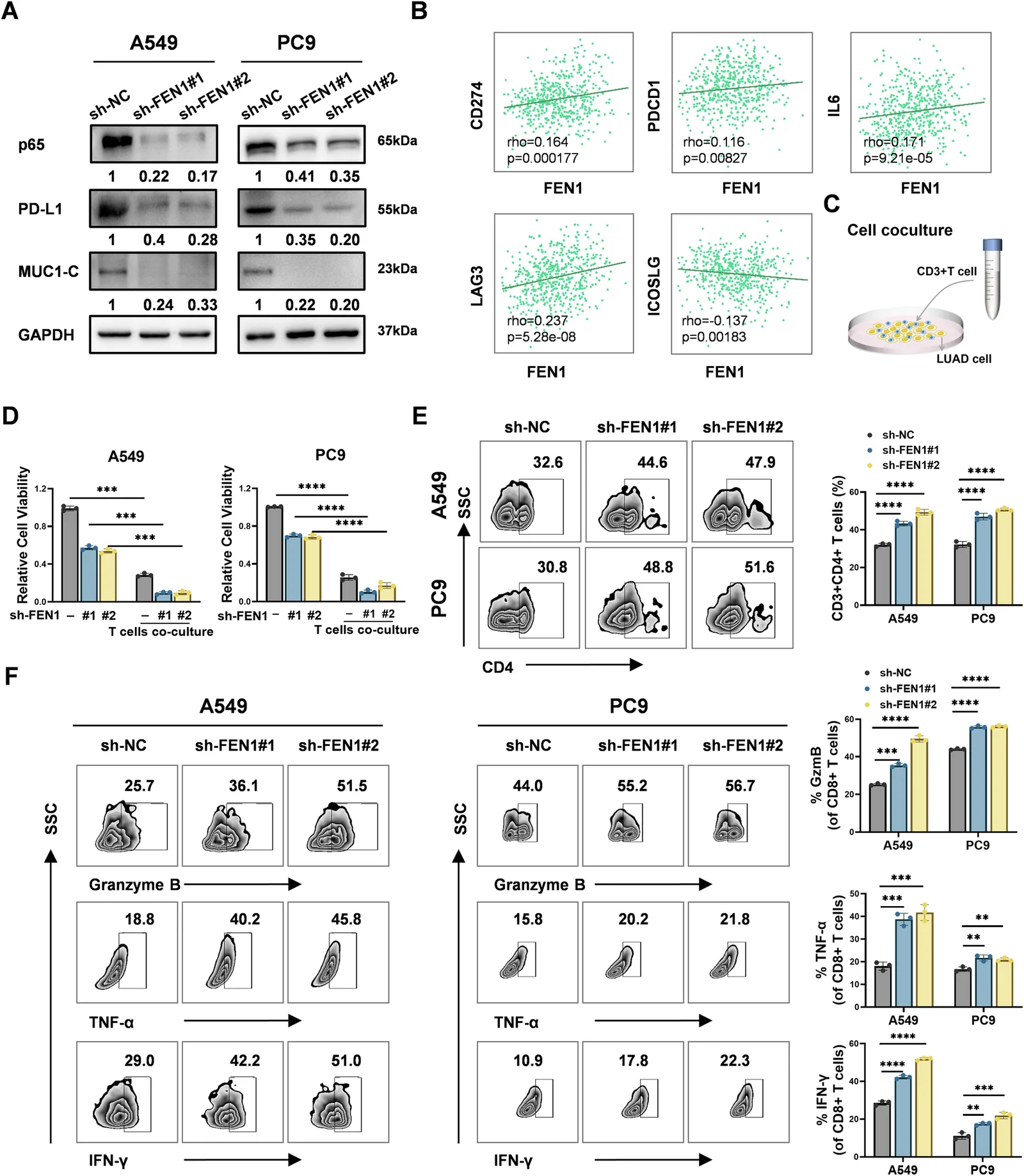
FEN1 downregulation alleviates LUAD-mediated immune suppression by increasing CD4^+^ T cells and enhancing CD8^+^ T cell function. **A** Western blot analysis detecting the expression of p65, PD-L1, and MUC1-C after FEN1 downregulation. **B** TIMER2.0 database analysis showing the correlation between FEN1 and PD-L1, PD-1, IL-6, LAG3, and ICOSLG. **C** Schematic diagram of co-culture of T cells with LUAD cells. **D** MTT assay determining the viability of LUAD cells after co-culture with T cells. **E** Flow cytometry analyzing the proportion of CD4^+^ T cells after co-culture of T cells with LUAD cells. **F** Flow cytometry detecting the intracellular expression of granzyme B, TNF-α, and IFN-γ in CD8^+^ T cells after co-culture of T cells with LUAD cells. Data are presented as means ± SD of three independent biological experiments. **P < 0.01, ***P < 0.001, ****P < 0.0001.

As a master oncoprotein, MUC1-C drives key hallmarks of cancer [20–22]. Critically, in LUAD, MUC1-C directly interacts with NF-κB p65 to enhance its promoter-binding activity, triggering two critical downstream effects: [1] driving MUC1-C transcription to form a persistent MUC1-C/p65 positive feedback loop; [2] sustaining PD-L1 transcription, which inhibits T-cell function and promotes immune evasion [23, 24]. To explore how FEN1 regulates tumor immunity via MUC1-C, we first examined the effect of FEN1 downregulation on these key molecules in LUAD cells. Our results showed that FEN1 downregulation significantly reduced p65 and PD-L1 expression in LUAD cells (Fig. 3A). Additionally, immune correlation analysis in LUAD revealed that FEN1 expression was positively correlated with programmed cell death 1(PDCD1/PD-1), cluster of differentiation 274(CD274/PD-L1), interleukin-6 (IL-6), and lymphocyte activation gene-3 (LAG-3), but negatively correlated with inducible T cell costimulator ligand (ICOSLG) (Fig. 3B). Herein, PD-1 and LAG-3 are key co-inhibitory molecules expressed on the surface of T cells. By binding to their respective ligands (PD-L1 for PD-1 and MHC class II molecules for LAG-3) on tumor cells, they transduce inhibitory signals into T cells, ultimately leading to T cell dysfunction [25, 26]. IL-6, a pleiotropic pro-inflammatory cytokine, plays a pivotal role in linking innate and adaptive immune responses by promoting the lineage-specific differentiation of naive CD4^+^ T cells [27]. Furthermore, ICOSLG serves as a critical molecule that upregulates and potentiates T cell-mediated immune responses [28]. Thus, these observations implied a functional link between FEN1 and T cell-mediated immunity, prompting the hypothesis that FEN1 downregulation enhances T cell function by reducing MUC1-C and p65 expression, thereby attenuating PD-L1-mediated immune suppression. Indeed, knockdown of FEN1 in tumor cells resulted in significantly reduced cell viability upon co-culture with T cells (Fig. 3C, D). To determine whether FEN1 influences the immunosuppressive capacity of tumor cells, we analyzed the subsets and functional status of T cell populations. The results showed that FEN1 downregulation in tumor cells significantly increased the proportion of CD4^+^ T cells (Fig. 3E) but did not affect that of CD8^+^ T cells (Fig. S5A). Furthermore, CD8^+^ T cells co-cultured with FEN1-downregulated tumor cells exhibited significantly higher intracellular expression of granzyme B, TNF-α, and IFN-γ (Fig. 3F), whereas the expression of TNF-α and IFN-γ in CD4^+^ T cells remained unaffected (Fig. S5B, C). These results indicated that FEN1 downregulation alleviates LUAD-mediated immune suppression by increasing CD4^+^ T cells and enhancing CD8^+^ T cell function.

**Fig. 4:**
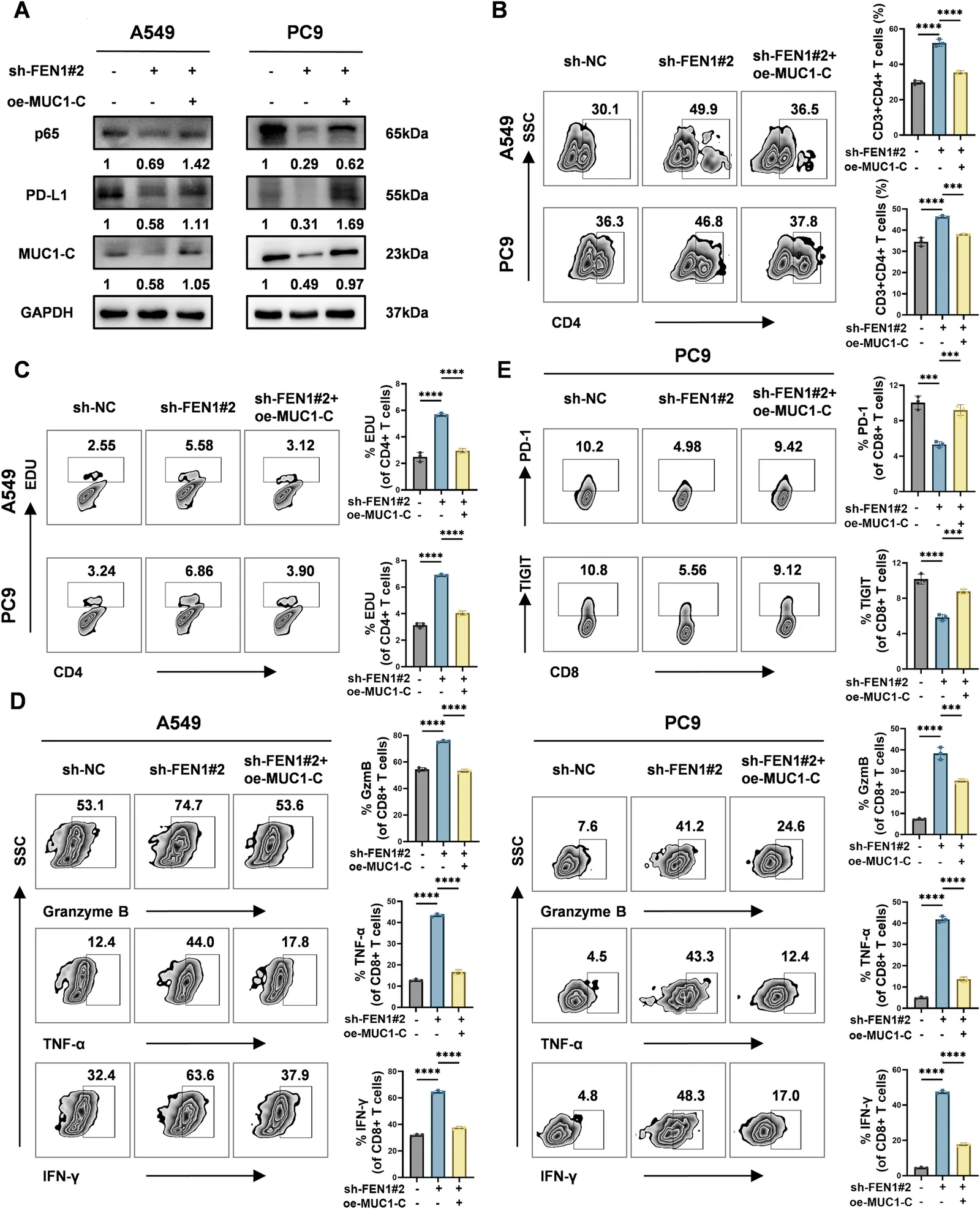
MUC1-C mediates the antitumor immunity induced by FEN1 downregulation. After ectopic overexpression of MUC1-C in FEN1-downregulated LUAD cells. **A** Western blot analysis detecting the expression of p65, PD-L1, and MUC1-C; **B** Flow cytometry analyzing the proportion of CD4^+^ T cells after co-culture of T cells with LUAD cells. **C** Representative flow cytometry images and quantitative analysis of EdU^+^ cells specifically gated within the CD4^+^ T cell population in the co-culture system. **D** Flow cytometry detecting the intracellular expression of granzyme B, TNF-α, and IFN-γ in CD8^+^ T cells after co-culture of T cells with LUAD cells. **E** Representative flow cytometry plots and quantitative analysis showing the percentage of PD-1^+^/ TIGIT^+^ cells specifically gated within the CD8^+^ T cell population following co-culture with PC9 cells under the indicated conditions. Data are presented as means ± SD of three independent biological experiments. ***P < 0.001, ****P < 0.0001.

To confirm MUC1-C as the mediator of these effects, we performed rescue experiments. MUC1-C overexpression effectively restored p65 and PD-L1 expression (Fig. 4A) and reduced the FEN1 knockdown-induced increase in CD4^+^ T cells without affecting the total proportion of CD8^+^ T cells (Fig. 4B, Fig. S6A). Mechanistically, in vitro assays showed that the FEN1/MUC1-C axis did not affect T cell chemotaxis (Fig. S6B). Instead, FEN1 knockdown increased the local proliferation (EdU^+^) of CD4^+^ T cells, which was reversed by MUC1-C overexpression (Fig. 4C). Therefore, the increased CD4^+^ T cell population is driven by local proliferation rather than infiltration. For CD8^+^ T cells, FEN1 knockdown increased the expression of effector molecules and decreased exhaustion markers (PD-1^+^ and TIGIT^+^), whereas MUC1-C overexpression reversed these phenotypes (Fig. 4D, E, Fig. S6C). Collectively, these findings establish MUC1-C as the primary mediator of the FEN1 downregulation-induced antitumor immunity.

**Fig. 5:**
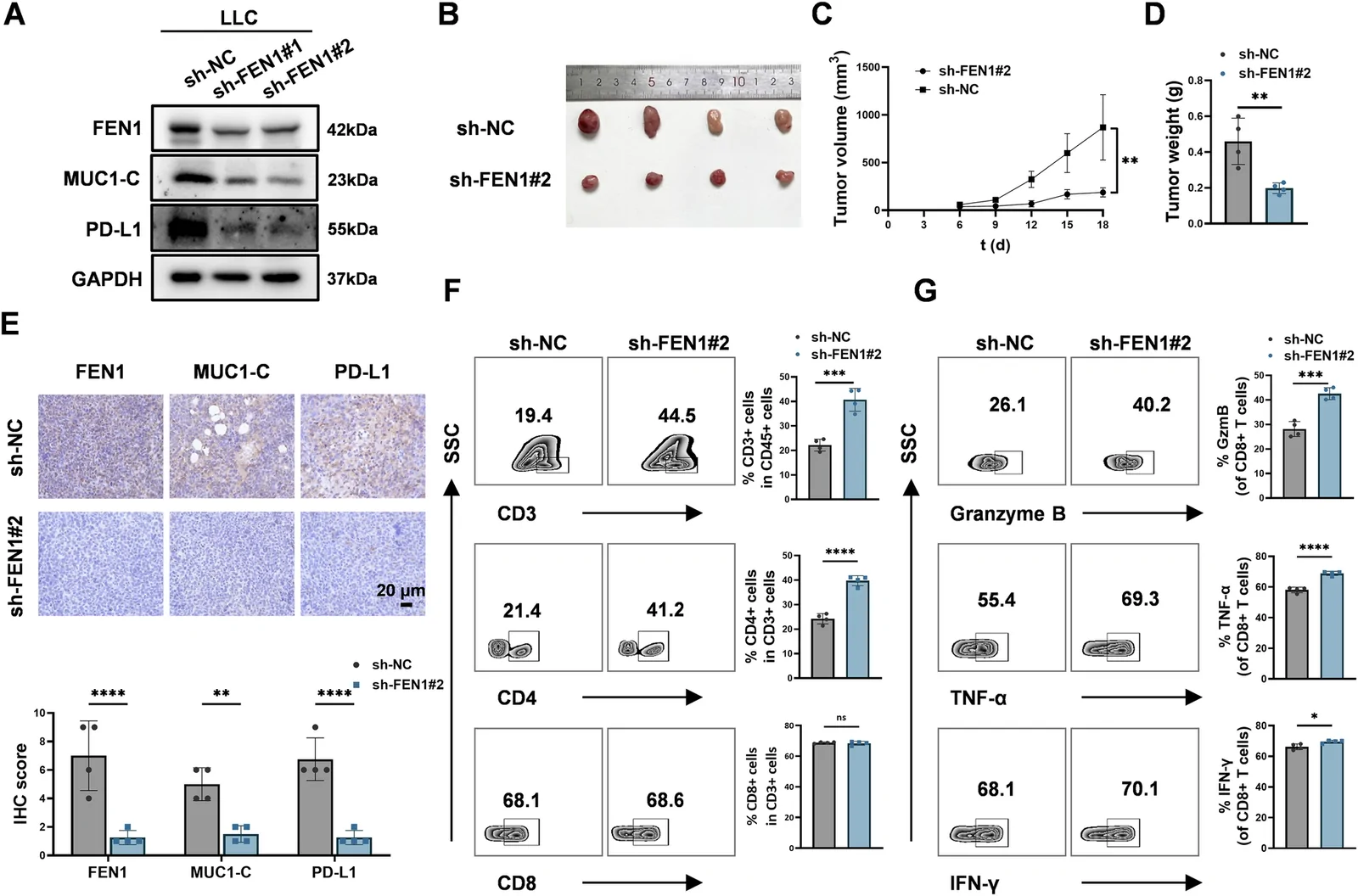
FEN1 downregulation boosts antitumor immunity by increasing intratumoral CD4^+^ T cells and enhancing CD8^+^ T cell function in vivo. **A** Western blot analysis verifying the knockdown efficiency of FEN1 and MUC1-C and PD-L1 protein expression in LLC stable cell lines. **B** Tumor photographs of the sh-NC group and sh-FEN1#2 group. **C** Tumor volumes of the sh-NC group and sh-FEN1#2 group. **D** Tumor weights of the sh-NC group and sh-FEN1#2 group. **E** Representative images and quantitative results of FEN1, MUC1-C, and PD-L1 IHC staining in tumor tissues. **F** Flow cytometry analyzing the proportions of intratumoral CD3^+^ T, CD4^+^ T, and CD8^+^ T cells. **G** Flow cytometry detecting the intracellular expression of granzyme B, TNF-α, and IFN-γ in intratumoral CD8^+^ T cells. Data are shown as means ± SD. *n* = 4, *P < 0.05, **P < 0.01, ***P < 0.001, ****P < 0.0001, ns, not significant.

For in vivo validation, we generated FEN1-stable knockdown mouse Lewis lung cancer (LLC) cells and established subcutaneous xenografts in C57BL/6 mice. In these knockdown cells, Western blotting showed downregulation of both MUC1-C and PD-L1 (Fig. 5A). Compared to controls, FEN1 knockdown significantly reduced tumor size, volume, and weight (Fig. 5B–D), and decreased FEN1, MUC1-C, and PD-L1 expression in tumor tissues (Fig. 5E). Single-cell suspension analysis of tumor tissues showed that FEN1 knockdown increased total CD3^+^ T cells and CD4^+^ T cells with no change in CD8^+^ T cells (Fig. 5F), and enhanced granzyme B, TNF-α, and IFN-γ expression in CD8^+^ T cells (Fig. 5G); CD4^+^ T cell cytokine levels were unaffected (Fig. S7A). These in vivo results confirmed that FEN1 downregulation boosts antitumor immunity by increasing intratumoral CD4^+^ T cells and enhancing CD8^+^ T cell function. In summary, our in vitro and in vivo results demonstrated that FEN1 downregulation suppresses the MUC1-C/p65/PD-L1 signaling axis, thereby alleviating tumor immunosuppression.

### FEN1 interacts with EIF4A3 and maintains its functional activity

**Fig. 6:**
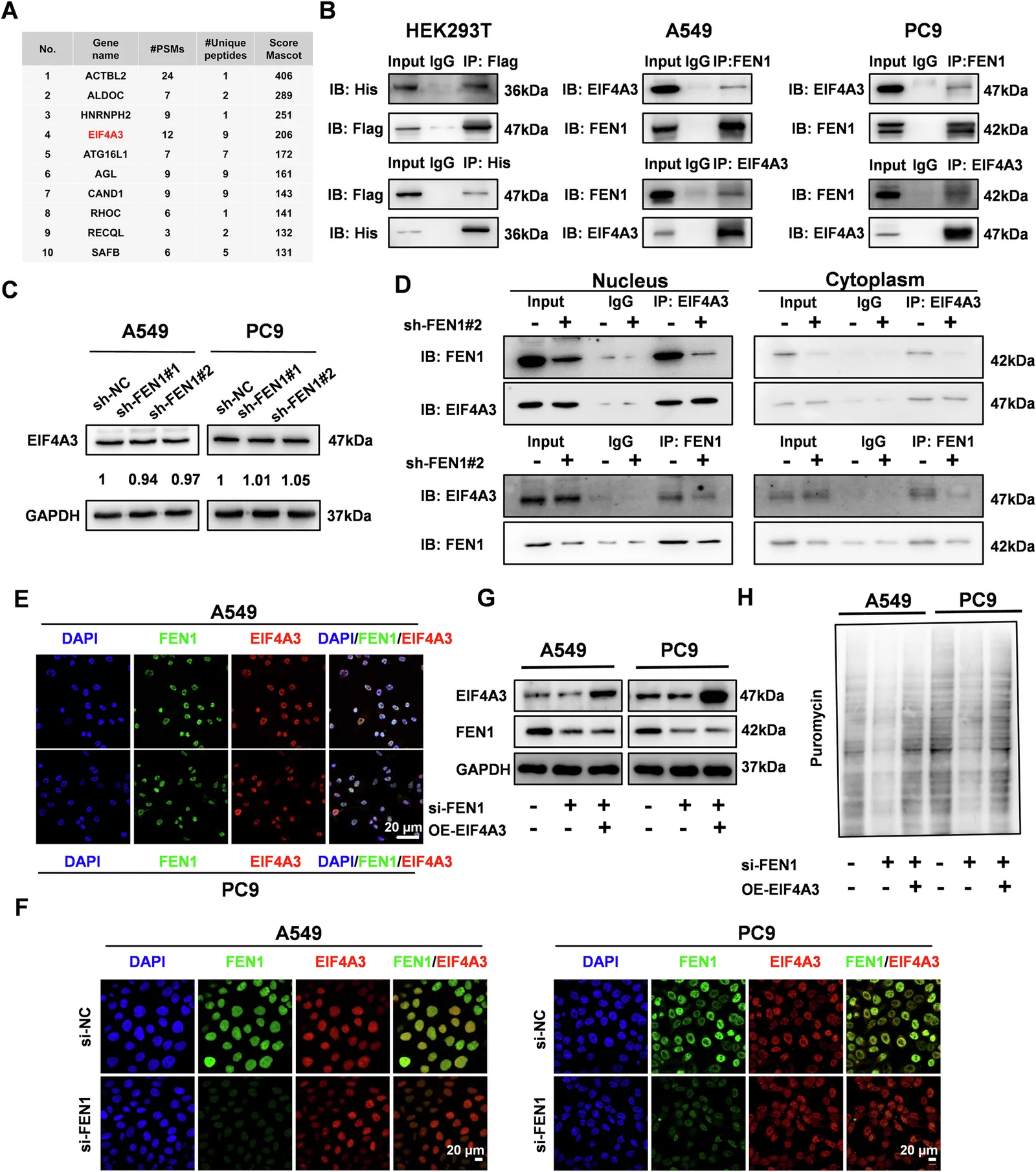
FEN1 interacts with EIF4A3 and maintains its functional activity. **A** Top 10 proteins interacting with FEN1 identified by IP-MS. **B** Co-IP assay verifying the interaction between FEN1 and EIF4A3 in HEK293T and LUAD cells. **C** Western blot analysis detecting the expression of EIF4A3 after FEN1 knockdown. **D** Endogenous Co-IP assays of nuclear and cytoplasmic fractions from A549 cells using anti-FEN1 and anti-EIF4A3 antibodies. **E** Immunofluorescence staining showing the colocalization of FEN1 and EIF4A3 in LUAD cells. **F** Immunofluorescence staining examining the colocalization of FEN1 and EIF4A3 in LUAD cells transfected with control or FEN1 small interfering RNA (siRNA). **G**, **H** Immunoblots of FEN1, EIF4A3 (G) and puromycin-labeled nascent proteins (H) in LUAD cells following FEN1 knockdown with or without EIF4A3 overexpression. Puromycin (5 μg/mL) was added for 30 min before harvest. All experiments were performed in biological triplicate.

To elucidate the molecular mechanism by which FEN1 regulates MUC1-C expression, we performed IP-MS on A549 cell lysates using an anti-FEN1 antibody and identified eukaryotic translation initiation factor 4A3 (EIF4A3), a multifaceted DEAD-box RNA helicase and core component of the exon junction complex (EJC) [29], as a potential FEN1-interacting protein (Fig. 6A). Given recent findings linking EIF4A3 to MUC1-C regulation in non-small cell lung cancer [30], we hypothesized that FEN1 modulates MUC1-C expression via EIF4A3.

We first sought to validate this interaction. Co-IP assays in HEK293T cells expressing exogenous Flag-FEN1 and His-EIF4A3, together with endogenous Co-IP in A549 and PC9 cells, confirmed their robust interaction (Fig. 6B). Recognizing the inherent nucleic acid-binding properties of both proteins, we further subjected the cell lysates to Benzonase treatment. The interaction persisted despite complete nucleic acid digestion, indicative of an interaction independent of DNA or RNA bridging (Fig. S8A). We subsequently investigated whether FEN1 modulates the protein expression or subcellular localization of EIF4A3. Western blot results showed that FEN1 knockdown did not change the total EIF4A3 protein level in LUAD cells (Fig. 6C). Nuclear and cytoplasmic fractionation was subsequently performed (Fig. S8B). Co-IP assays from the separated fractions revealed that the FEN1-EIF4A3 complex is predominantly formed in the nucleus (Fig. 6D, Fig. S8C), a finding corroborated by their colocalization in LUAD cells and lung cancer tissues (Fig. 6E, Fig. S8D). Notably, while FEN1 depletion effectively disrupted this interaction (Fig. 6D, F, Fig. S8C), both the total protein expression and subcellular localization of EIF4A3 remained unchanged, without aberrant cytoplasmic translocation (Fig. 6D, F, Fig. S8C).

Having excluded alterations in EIF4A3 protein abundance and spatial distribution, we reasoned that the interaction with FEN1 is indispensable for maintaining EIF4A3’s functional activity. Considering that EIF4A3 orchestrates multiple steps of mRNA maturation and translation [29], we assessed this functional impact at a broader cellular level by measuring global protein synthesis using the SUnSET assay. Cells transfected with FEN1 siRNA exhibited a significant reduction in puromycin incorporation compared to control cells, indicating a decrease in global translation (Fig. 6G, H). Notably, ectopic EIF4A3 overexpression successfully rescued the puromycin incorporation levels in FEN1-KD cells (Fig. 6G, H). These findings indicated that FEN1 regulates global protein synthesis by sustaining EIF4A3 functional activity. Altogether, these data demonstrated that FEN1 interacts with EIF4A3 and is essential for maintaining its functional activity.

### FEN1 anchors the EIF4A3 N-terminus independent of its nuclease activity to promote MUC1-C mRNA stability and translation efficiency

**Fig. 7:**
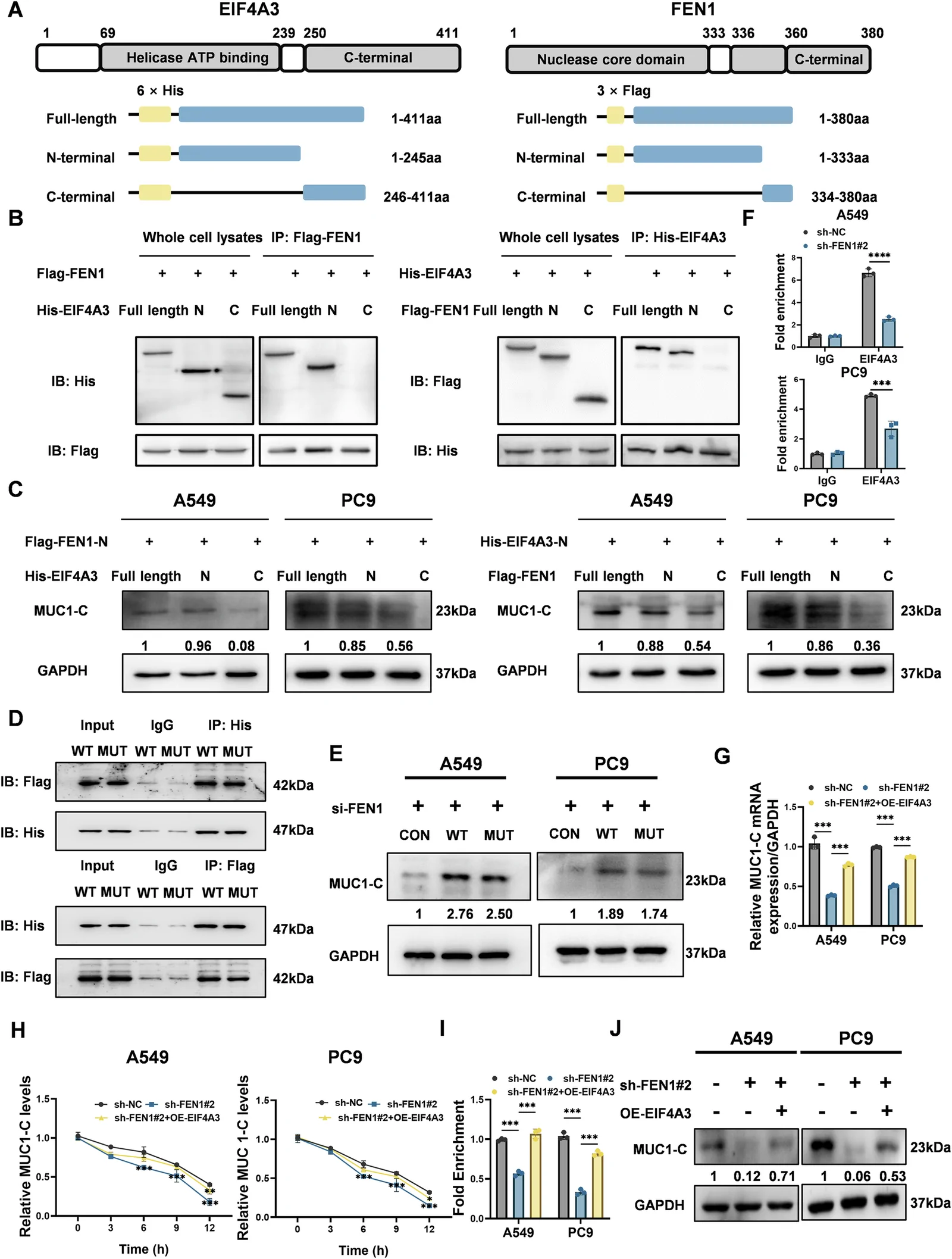
FEN1 anchors the EIF4A3 N-terminus independent of its nuclease activity to promote MUC1-C mRNA stability and translation efficiency. **A** Schematic diagrams of EIF4A3 and FEN1 truncation mutants. **B** Co-IP assay identifying the interacting domains between EIF4A3 and FEN1 in HEK293T cells. **C** Western blot analysis detecting the effects of transfected EIF4A3 and FEN1 truncation mutants on the expression of MUC1-C in LUAD cells. **D** Exogenous Co-IP assays in HEK293T cells co-transfected with His-EIF4A3 and either Flag-FEN1-WT (WT) or Flag-FEN1-D181A (MUT). **E** Western blot analysis of MUC1-C protein expression in FEN1-knockdown LUAD cells rescued with Flag-FEN1-WT (WT) or Flag-FEN1-D181A (MUT). **F** RIP-qPCR assay investigating the impact of FEN1 downregulation on EIF4A3-enriched MUC1-C mRNA in LUAD cells. **G** RT-qPCR analysis of basal MUC1-C mRNA levels in indicated groups. **H** Act D chase assay measuring the relative MUC1-C mRNA remaining at the indicated time points. **I** Ribo-RIP-qPCR analysis showing the relative ribosome occupancy of MUC1-C mRNA. Data are normalized to input RNA. **J** Western blot analysis of MUC1-C protein expression across the three experimental groups. All experiments were performed in biological triplicate. Data are shown as means ± SD. ***P < 0.001, ****P < 0.0001.

To map the interacting domains of FEN1 and EIF4A3, we generated N-terminal or C-terminal truncation mutants of both proteins (Fig. 7A). These mutants were transfected into HEK293T cells, and their expression was confirmed via anti-Flag and anti-His immunoblotting (Fig. 7B). Co-IP assays in HEK293T cells co-transfected with Flag-FEN1 (full-length or truncation mutants) and His-EIF4A3 (full-length or truncation mutants) revealed that the N-terminus of EIF4A3 interacts with the N-terminus of FEN1 (Fig. 7B). To validate whether this N-terminal interaction modulates MUC1-C expression, we performed functional assays in LUAD cells. In cells expressing the Flag-FEN1 N-terminal fragment, co-expression of the His-EIF4A3 C-terminal fragment resulted in a significant downregulation of MUC1-C, whereas the full-length or N-terminal His-EIF4A3 maintained high MUC1-C levels (Fig. 7C). Similarly, in the presence of the His-EIF4A3 N-terminal fragment, co-expression of the FEN1 C-terminal fragment, but not the full-length or N-terminal FEN1, led to a reduction in MUC1-C expression (Fig. 7C). These findings demonstrate that the N-terminal interaction between FEN1 and EIF4A3 is critical for maintaining EIF4A3 functional activity and MUC1-C expression.

To determine whether the regulation of MUC1-C depends on the nuclease activity of FEN1 or its interaction with EIF4A3, we generated the nuclease-dead mutant Flag-FEN1-D181A [31]. Co-IP assays in HEK293T cells confirmed that the D181A mutation does not disrupt the interaction between FEN1 and EIF4A3, maintaining binding at levels comparable to wild-type FEN1 (Fig. 7D). Next, we performed functional rescue experiments in FEN1-knockdown LUAD cells. Ectopic expression of either wild-type Flag-FEN1 or the Flag-FEN1-D181A mutant restored MUC1-C protein levels to a similar extent (Fig. 7E). These results suggest that the interaction with EIF4A3, rather than the nuclease activity of FEN1, is fundamentally required for MUC1-C expression.

To elucidate the consequences of this structural complex on specific RNA targets, we first assessed its mRNA-binding capacity. Mechanistically, RIP-qPCR demonstrated that FEN1 downregulation impaired the direct binding of EIF4A3 to MUC1-C mRNA (Fig. 7F), suggesting that FEN1 is required for the efficient deposition of EIF4A3 onto its target mRNAs. Given that a canonical function of EIF4A3 is to govern the stability and translation efficiency of its bound transcripts [32], we hypothesized that this impaired recruitment would critically disrupt downstream post-transcriptional dynamics. Indeed, RT-qPCR analysis revealed that FEN1 knockdown significantly reduced basal MUC1-C mRNA levels, while functional EIF4A3 overexpression effectively restored transcript abundance (Fig.7G). Act D chase assays further showed that FEN1 depletion markedly accelerated MUC1-C mRNA degradation, a phenotype that was successfully rescued by EIF4A3 overexpression (Fig. 7H). To explore post-transcriptional regulation beyond mRNA stability, we performed a Ribo-RIP assay to assess translation efficiency. The active ribosome occupancy of MUC1-C mRNA was significantly reduced in FEN1-KD cells, but was efficiently restored upon EIF4A3 overexpression (Fig.7I). Consistent with these mRNA and translational dynamics, Western blot analysis confirmed that the reduction in MUC1-C protein levels caused by FEN1 knockdown was reversed by ectopic EIF4A3 expression (Fig. 7J). These integrated data demonstrated that FEN1 depletion impairs EIF4A3-mediated maintenance of the stability and translation of MUC1-C mRNA. Overall, these results revealed that FEN1 structurally anchors the N-terminus of EIF4A3 to facilitate its binding to MUC1-C mRNA, thereby synergistically promoting MUC1-C transcript stability and translation efficiency.

### Expression, correlation, and prognostic significance of the FEN1/MUC1-C/PD-L1 axis in clinical specimens

**Fig. 8:**
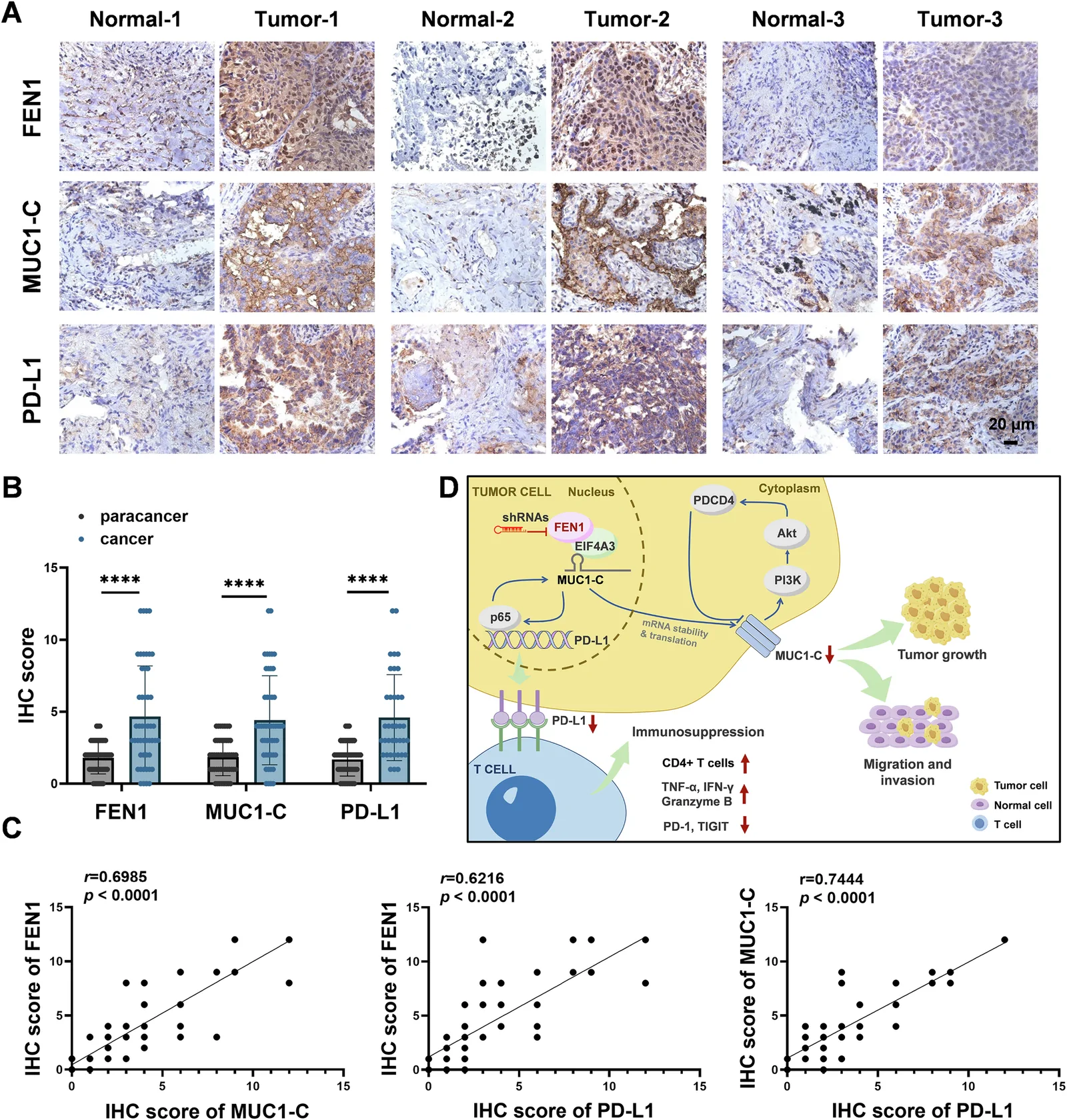
Expression and correlation analysis of FEN1, MUC1-C, and PD-L1 in clinical specimens. **A** IHC detection of FEN1, MUC1-C, and PD-L1 expression in normal tissues and LUAD tissues from clinical specimens. **B** Quantitative results of FEN1, MUC1-C, and PD-L1 expression in normal tissues and LUAD tissues. **C** Correlation analysis of FEN1, MUC1-C, and PD-L1 expression in LUAD tissues. **D** Schematic model illustrating a novel FEN1/EIF4A3/MUC1-C signaling axis driving LUAD progression and immunosuppression. FEN1 knockdown disrupts its interaction with EIF4A3, reducing MUC1-C mRNA stability and translation. Decreased MUC1-C inhibits PI3K/AKT signaling (suppressing tumor growth, migration, and invasion) and p65/PD-L1-mediated immunosuppression. The latter leads to enhanced anti-tumor immunity, with expanded CD4^+^ T cells, restored CD8^+^ T cell function (increased TNF-α, IFN-γ, Granzyme B), and reduced PD-1/TIGIT expression. Data are shown as means ± SD. *n* = 49, ****P < 0.0001.

We next evaluated the expression of FEN1, MUC1-C, and PD-L1 in a clinical cohort of 49 LUAD patients using IHC on paired tumor and adjacent non-tumor tissues. Quantitative analysis revealed that the protein levels of FEN1, MUC1-C, and PD-L1 were all significantly higher in tumor specimens than in adjacent tissues (Fig. 8A, B). Moreover, Spearman’s correlation analysis demonstrated strong positive pairwise correlations among the expression of these three proteins within the tumors (Fig. 8C). The analysis of the TCGA-LUAD dataset confirmed that this correlation was independent of EGFR mutation status, remaining significant in EGFR-wild-type patients (Fig. S9A) and showing a consistent trend in EGFR-mutant patients (Fig. S9B). Kaplan–Meier survival analysis indicated that patients with high dual expression of FEN1 and MUC1-C had a significantly unfavorable prognosis (Fig. S9C). Furthermore, this trend remained consistent for the entire axis, as patients with high co-expression of FEN1, MUC1-C, and PD-L1 also exhibited significantly poorer overall survival than those with low co-expression (Fig. S9D).

Together, these clinical findings demonstrated that the FEN1/MUC1-C/PD-L1 axis is coordinately activated in LUAD, providing significant prognostic value and clinical relevance that potentially extends across different EGFR mutation backgrounds.

## Discussion

FEN1 is a pivotal enzyme in DNA replication and repair, whose oncogenic role has been established in several malignancies, including breast cancer, HCC, cholangiocarcinoma, gastric cancer and prostate cancer [8, 9, 33–35]. Although the contribution of FEN1 to non-small cell lung cancer (NSCLC) cell proliferation and cisplatin resistance has been reported, previous studies have primarily focused on its role in DNA damage repair [36]. Consequently, the broader molecular mechanisms, particularly its intersection with oncogenic signaling and tumor immunity, remain incompletely understood. In this study, we unveil a novel FEN1/MUC1-C axis that governs both the malignant phenotypes and immune evasion capabilities of LUAD cells, bearing strong prognostic significance in clinical cohorts.

Our findings identify MUC1-C as a critical downstream effector of FEN1 in LUAD. We demonstrate that FEN1 knockdown disrupts the MUC1-C/PI3K/AKT autoregulatory loop, a driver of oncogenesis. This disruption recapitulates the well-established effects of FEN1 downregulation, namely the inhibition of cancer cell proliferation, migration, and invasion [8, 9, 33]. Importantly, it uncovers a previously unappreciated role: the induction of cellular senescence. The induction of senescence without concurrent apoptosis is a notable observation, potentially reflecting the phenomenon of apoptosis resistance in senescent cells [37]. This concept is exemplified by the findings that inhibition of the integrin α6β4/Src/AKT signaling pathway induces senescence by antagonizing apoptosis in NSCLC cells [38]. The finding is also consistent with our previous work in breast cancer, where inhibiting the FEN1-PBX1 axis induced senescence via ROS accumulation [12]. Together, these observations suggest a conserved pro-senescence function for FEN1 inhibition across cancer types.

Beyond its intrinsic oncogenic effects, the FEN1/MUC1-C axis is a critical regulator of the anti-tumor immune response. While MUC1-C is known to foster an immunosuppressive microenvironment in cancers like triple-negative breast cancer [39], its role in mediating FEN1-driven immune evasion in LUAD remains undefined. Our data uncover that FEN1 downregulation robustly enhances anti-tumor immunity, evidenced by increased CD4^+^ T cell infiltration and amplified CD8^+^ T cell effector function both in vitro and in vivo. Mechanistically, this immunostimulatory phenotype is orchestrated through MUC1-C. Rescue experiments demonstrated that MUC1-C overexpression restored p65 and PD-L1 levels and effectively nullified the T cell activation induced by FEN1 knockdown, a finding corroborated in our Lewis lung carcinoma model. This work significantly advances upon previous studies that merely correlated FEN1 with PD-L1 expression in HNSCC and OSCC without elucidating the governing mechanism or providing in vivo validation [13, 14]. Importantly, our clinical cohort analysis further consolidates these findings by demonstrating strong positive pairwise correlations and prognostic relevance of the FEN1/MUC1-C/PD-L1 axis across diverse EGFR mutational backgrounds. We therefore conclude that FEN1 downregulation mediates anti-tumor immunity by inhibiting the MUC1-C/p65/PD-L1 axis in LUAD.

To further elucidate the upstream regulation, we investigated how FEN1 controls MUC1-C expression. IP-MS identified EIF4A3 as a potential interacting partner of FEN1. Although EIF4A3 is predominantly known as an RNA-binding protein, its role as a protein-protein interaction hub is increasingly recognized. For instance, EIF4A3 has been shown to bind IFN regulatory factor 3 to suppress antiviral immunity [40] and to interact with Flotillin-1 to promote lung cancer progression [41]. In line with these precedents, we confirmed a nucleic-acid-independent interaction between FEN1 and EIF4A3 predominantly in the nucleus. Functionally, we found that FEN1 knockdown did not alter EIF4A3 protein levels or its nuclear distribution, but paralyzed its functional activity, leading to a profound decrease in global translation. We mapped this interaction to the N-termini of both proteins, and our mechanistic validations utilizing the nuclease-dead FEN1-D181A mutant definitively uncoupled this non-canonical function from FEN1’s canonical role in DNA repair. Structurally, the N-terminus of EIF4A3 encompasses its highly conserved ATP-binding domain, which is essential for its RNA clamp function and enzymatic activity [42]. We postulate that FEN1 docking onto this critical region stabilizes EIF4A3 in an active conformation. Indeed, disrupting this interaction by utilizing truncation mutants significantly downregulated MUC1-C. RIP-qPCR, Act D chase, and Ribo-RIP analyses collectively established that FEN1 depletion specifically impairs the direct binding of EIF4A3 to MUC1-C mRNA, thereby disrupting EIF4A3-mediated maintenance of transcript stability and translation efficiency. While our data strongly align with the functional failure of EIF4A3 in executing its canonical post-transcriptional roles (such as EJC nucleation), we note that EIF4A3 is a versatile DEAD-box RNA helicase. It is highly plausible that FEN1 also stabilizes its intrinsic helicase activity, facilitating the unwinding of complex secondary structures in the target mRNAs to promote ribosome scanning. This mechanistic insight aligns with and extends previous reports that the related helicase EIF4A regulates MUC1-C translation [19], establishing the FEN1/EIF4A3/MUC1-C axis as a novel regulatory pathway in LUAD.

In this study, we demonstrated that FEN1 promotes LUAD progression primarily by regulating MUC1-C. To evaluate the translational potential of our findings, we directly targeted MUC1 using the specific MUC1-C peptide inhibitor GO-203. Consistent with the phenotypes observed upon FEN1 knockdown, pharmacological inhibition of MUC1 significantly suppressed malignant behaviors (proliferation, migration, and invasion), triggered cellular senescence, and attenuated PD-L1-mediated immune signaling (Fig.S10). These data phenocopy the effects of FEN1 depletion, highlighting that functionally blocking the downstream effector of the FEN1/MUC1-C axis represents a viable and promising therapeutic strategy for LUAD.

Nevertheless, several limitations should be noted. First, while we identified that the N-termini of FEN1 and EIF4A3 mediate their interaction, the critical amino acid residues involved remain to be elucidated. Second, although our work establishes a strong preclinical rationale for targeting the FEN1/MUC1-C axis, the therapeutic potential of a FEN1 inhibitor, particularly in combination with anti-PD-1/PD-L1 immunotherapies, was not investigated in the current study. Recent studies indicate that the efficacy of immunotherapy is largely influenced by complex interactions within the tumor microenvironment (TME), where specific tumor-associated factors and cytokines promote immunosuppression [43, 44]. Our findings suggest that FEN1 depletion modulates the TME toward a more immune-active state by promoting local CD4^+^ T cell expansion and restoring CD8^+^ T cell function. Therefore, future investigations evaluating whether FEN1 blockade synergizes with immune checkpoint blockade or other TME-modulating agents will be valuable for optimizing therapeutic outcomes in LUAD.

In conclusion, this study unveils a novel FEN1/EIF4A3/MUC1-C signaling axis that drives LUAD progression through a dual-pronged mechanism. We demonstrate that FEN1 anchors the N-terminus of EIF4A3 independent of its nuclease activity, thereby sustaining MUC1-C mRNA stability and translation efficiency. Downregulation of FEN1 disrupts this interaction, leading to suppressed MUC1-C expression. Intrinsically, this disruption blocks the MUC1-C/PI3K/AKT autoregulatory loop, inhibiting cell proliferation and metastasis. Extrinsically, it impairs the MUC1-C/p65/PD-L1 immune checkpoint axis, thereby reactivating anti-tumor immunity. Clinically, FEN1, MUC1-C, and PD-L1 are coordinately upregulated in LUAD tissues, and their co-expression predicts poor patient survival regardless of EGFR mutation status. Our findings reveal a direct molecular link between FEN1, post-transcriptional control, and immune evasion, providing a strong rationale for co-targeting malignant progression and the immunosuppressive microenvironment in LUAD (Fig. 8D).

## Supplementary information

Supplemental Information

original WB bands

Proteomics data

## Data Availability

The datasets presented in this study can be found in public online repositories. The proteomics data have been deposited to the ProteomeXchange Consortium (https://proteomecentral.proteomexchange.org) via the iProX partner repository [45, 46] with the dataset identifier PXD078203. All other data associated with this study are present in the paper or the supplementary material.

## References

[1] Luo G, Zhang Y, Rumgay H, Morgan E, Langselius O, Vignat J, et al. Estimated worldwide variation and trends in incidence of lung cancer by histological subtype in 2022 and over time: a population-based study. Lancet Respir Med. 2025;13:348–63. 10.1016/S2213-2600(24)00428-4 39914442

[2] Howlader N, Forjaz G, Mooradian MJ, Meza R, Kong CY, Cronin KA, et al. The Effect of Advances in Lung-Cancer Treatment on Population Mortality. N Engl J Med. 2020;383:640–9. 10.1056/NEJMoa1916623 PMC8577315 32786189

[3] Ettinger DS, Wood DE, Aisner DL, Akerley W, Bauman JR, Bharat A, et al. NCCN Guidelines® Insights: Non-Small Cell Lung Cancer, Version 2.2023. J Natl Compr Canc Netw. 2023;21:340–50. 10.6004/jnccn.2023.0020 37015337

[4] Meyer ML, Fitzgerald BG, Paz-Ares L, Cappuzzo F, Jänne PA, Peters S, et al. New promises and challenges in the treatment of advanced non-small-cell lung cancer. Lancet. 2024;404:803–22. 10.1016/S0140-6736(24)01029-8 39121882

[5] Zhang Q, Shen Y, Che Y, Jia L, Xiao X, Xu H, et al. Hypoxia-inducible APCDD1L-AS1 promotes osimertinib resistance by stabilising DLST to drive tricarboxylic acid cycle in lung adenocarcinoma. J Exp Clin Cancer Res. 2025;44:197. 10.1186/s13046-025-03462-z PMC12239447 40634956

[6] Zheng L, Jia J, Finger LD, Guo Z, Zer C, Shen B. Functional regulation of FEN1 nuclease and its link to cancer. Nucleic Acids Res. 2011;39:781–94. 10.1093/nar/gkq884 PMC3035468 20929870

[7] Zheng L, Dai H, Zhou M, Li M, Singh P, Qiu J, et al. Fen1 mutations result in autoimmunity, chronic inflammation and cancers. Nat Med. 2007;13:812–9. 10.1038/nm1599 17589521

[8] Bian S, Ni W, Zhu M, Zhang X, Qiang Y, Zhang J, et al. Flap endonuclease 1 Facilitated Hepatocellular Carcinoma Progression by Enhancing USP7/MDM2-mediated P53 Inactivation. Int J Biol Sci. 2022;18:1022–38. 10.7150/ijbs.68179 PMC8771828 35173534

[9] Yuwei X, Bingzi D, Zhaowei S, Yujie F, Wei Z, Kun L, et al. FEN1 promotes cancer progression of cholangiocarcinoma by regulating the Wnt/β-catenin signaling pathway. Dig Liver Dis. 2024;56:695–704. 10.1016/j.dld.2023.08.050 37648642

[10] Yang F, Hu Z, Guo Z Small-Molecule Inhibitors Targeting FEN1 for Cancer Therapy. Biomolecules. 2022;12. 10.3390/biom12071007 PMC9312813 35883563

[11] Lu X, Liu R, Wang M, Kumar AK, Pan F, He L, et al. MicroRNA-140 impedes DNA repair by targeting FEN1 and enhances chemotherapeutic response in breast cancer. Oncogene. 2020;39:234–47. 10.1038/s41388-019-0986-0 31471584

[12] Wu M, Wu B, Huang X, Wang Z, Zhu M, Zhu Y, et al. Inhibition of the FEN1-PBX1 axis elicits cellular senescence in breast cancer via the increased intracellular reactive oxygen species levels. J Transl Med. 2025;23:248. 10.1186/s12967-025-06216-9 PMC11871692 40022092

[13] Wang S, Wang X, Sun J, Yang J, Wu D, Wu F, et al. Down-regulation of DNA key protein-FEN1 inhibits OSCC growth by affecting immunosuppressive phenotypes via IFN-γ/JAK/STAT-1. Int J Oral Sci. 2023;15:17. 10.1038/s41368-023-00221-8 PMC10130046 37185662

[14] Wang X, Xu S, Fu T, Wu Y, Sun W. Combination of downregulating FEN1 and PD-1 blockade enhances antitumor activity of CD8+ T cells against HNSCC cells in vitro. J Oral Pathol Med. 2023;52:834–42. 10.1111/jop.13485 37728572

[15] Zhao Y, Gu S, Li L, Zhao R, Xie S, Zhang J, et al. A novel risk signature for predicting brain metastasis in patients with lung adenocarcinoma. Neuro Oncol. 2023;25:2207–20. 10.1093/neuonc/noad115 PMC10708939 37379245

[16] Hattrup CL, Gendler SJ. Structure and function of the cell surface (tethered) mucins. Annu Rev Physiol. 2008;70:431–57. 10.1146/annurev.physiol.70.113006.100659 17850209

[17] Rajabi H, Kufe D. MUC1-C Oncoprotein Integrates a Program of EMT, Epigenetic Reprogramming and Immune Evasion in Human Carcinomas. Biochim Biophys Acta Rev Cancer. 2017;1868:117–22. 10.1016/j.bbcan.2017.03.003 PMC5548633 28302417

[18] Xu H, Du Z, Li Z, Liu X, Li X, Zhang X, et al. MUC1-EGFR crosstalk with IL-6 by activating NF-κB and MAPK pathways to regulate the stemness and paclitaxel-resistance of lung adenocarcinoma. Ann Med. 2024;56:2313671. 10.1080/07853890.2024.2313671 PMC10851807 38325364

[19] Jin C, Rajabi H, Rodrigo CM, Porco JA Jr, Kufe D. Targeting the eIF4A RNA helicase blocks translation of the MUC1-C oncoprotein. Oncogene. 2013;32:2179–88. 10.1038/onc.2012.236 PMC3443512 22689062

[20] Li W, Zhang N, Jin C, Long MD, Rajabi H, Yasumizu Y, et al. MUC1-C drives stemness in progression of colitis to colorectal cancer. JCI Insight. 2020;5. 10.1172/jci.insight.137112 PMC7406273 32427590

[21] Yasumizu Y, Rajabi H, Jin C, Hata T, Pitroda S, Long MD, et al. MUC1-C regulates lineage plasticity driving progression to neuroendocrine prostate cancer. Nat Commun. 2020;11:338. 10.1038/s41467-019-14219-6 PMC6969104 31953400

[22] Haratake N, Ozawa H, Morimoto Y, Yamashita N, Daimon T, Bhattacharya A, et al. MUC1-C Is a Common Driver of Acquired Osimertinib Resistance in NSCLC. J Thorac Oncol. 2024;19:434–50. 10.1016/j.jtho.2023.10.017 PMC10939926 37924972

[23] Ahmad R, Raina D, Joshi MD, Kawano T, Ren J, Kharbanda S, et al. MUC1-C oncoprotein functions as a direct activator of the nuclear factor-kappaB p65 transcription factor. Cancer Res. 2009;69:7013–21. 10.1158/0008-5472.CAN-09-0523 PMC2760979 19706766

[24] Bouillez A, Rajabi H, Jin C, Samur M, Tagde A, Alam M, et al. MUC1-C integrates PD-L1 induction with repression of immune effectors in non-small-cell lung cancer. Oncogene. 2017;36:4037–46. 10.1038/onc.2017.47 PMC5509481 28288138

[25] Dobosz P, Dzieciątkowski T. The Intriguing History of Cancer Immunotherapy. Front Immunol. 2019;10:2965. 10.3389/fimmu.2019.02965 PMC6928196 31921205

[26] Shi AP, Tang XY, Xiong YL, Zheng KF, Liu YJ, Shi XG, et al. Immune Checkpoint LAG3 and Its Ligand FGL1 in Cancer. Front Immunol. 2021;12:785091. 10.3389/fimmu.2021.785091 PMC8801495 35111155

[27] Szántó M, Yélamos J, Bai P. Specific and shared biological functions of PARP2 - is PARP2 really a lil’ brother of PARP1? Expert Rev Mol Med. 2024;26:e13. 10.1017/erm.2024.14 PMC11140550 38698556

[28] Iwata R, Hyoung Lee J, Hayashi M, Dianzani U, Ofune K, Maruyama M, et al. ICOSLG-mediated regulatory T-cell expansion and IL-10 production promote progression of glioblastoma. Neuro Oncol. 2020;22:333–44. 10.1093/neuonc/noz204 PMC7442411 31634400

[29] Kanellis DC, Espinoza JA, Zisi A, Sakkas E, Bartkova J, Katsori AM, et al. The exon-junction complex helicase eIF4A3 controls cell fate via coordinated regulation of ribosome biogenesis and translational output. Sci Adv. 2021;7. 10.1126/sciadv.abf7561 PMC8336962 34348895

[30] Takahashi S, Noro R, Seike M, Zeng C, Matsumoto M, Yoshikawa A, et al. Long Non-Coding RNA CRNDE Is Involved in Resistance to EGFR Tyrosine Kinase Inhibitor in EGFR-Mutant Lung Cancer via eIF4A3/MUC1/EGFR Signaling. Int J Mol Sci. 2021;22. 10.3390/ijms22084005 PMC8070547 33924522

[31] Shen B, Nolan JP, Sklar LA, Park MS. Functional analysis of point mutations in human flap endonuclease-1 active site. Nucleic Acids Res. 1997;25:3332–8. 10.1093/nar/25.16.3332 PMC146887 9241249

[32] Ye J, She X, Liu Z, He Z, Gao X, Lu L, et al. Eukaryotic Initiation Factor 4A-3: A Review of Its Physiological Role and Involvement in Oncogenesis. Front Oncol. 2021;11:712045. 10.3389/fonc.2021.712045 PMC8386015 34458150

[33] Wu M, Huang X, Wu B, Zhu M, Zhu Y, Yu L, et al. The endonuclease FEN1 mediates activation of STAT3 and facilitates proliferation and metastasis in breast cancer. Mol Biol Rep. 2024;51:553. 10.1007/s11033-024-09524-3 38642158

[34] Zhao E, Zhou C, Chen S. Flap endonuclease 1 (FEN1) as a novel diagnostic and prognostic biomarker for gastric cancer. Clin Res Hepatol Gastroenterol. 2021;45:101455. 10.1016/j.clinre.2020.04.019 32505732

[35] Wang Z, Yong C, Fu Y, Sun Y, Guo Z, Liu SB, et al. Inhibition of FEN1 promotes DNA damage and enhances chemotherapeutic response in prostate cancer cells. Med Oncol. 2023;40:242. 10.1007/s12032-023-02110-w 37452976

[36] He L, Luo L, Zhu H, Yang H, Zhang Y, Wu H, et al. FEN1 promotes tumor progression and confers cisplatin resistance in non-small-cell lung cancer. Mol Oncol. 2017;11:640–54. 10.1002/1878-0261.12058 PMC5467497 28371273

[37] Childs BG, Baker DJ, Kirkland JL, Campisi J, van Deursen JM. Senescence and apoptosis: dueling or complementary cell fates? EMBO Rep. 2014;15:1139–53. 10.15252/embr.201439245 PMC4253488 25312810

[38] Jung SH, Lee M, Park HA, Lee HC, Kang D, Hwang HJ, et al. Integrin α6β4-Src-AKT signaling induces cellular senescence by counteracting apoptosis in irradiated tumor cells and tissues. Cell Death Differ. 2019;26:245–59. 10.1038/s41418-018-0114-7 PMC6329762 29786073

[39] Yamashita N, Long M, Fushimi A, Yamamoto M, Hata T, Hagiwara M, et al. MUC1-C integrates activation of the IFN-γ pathway with suppression of the tumor immune microenvironment in triple-negative breast cancer. J Immunother Cancer. 2021;9:e002115. 10.1136/jitc-2020-002115 PMC7839859 33495298

[40] Gao Q, Jiang M, Zhao Y, Li G, Yang C, Ren C, et al. eIF4A3 Promotes RNA Viruses’ Replication by Inhibiting Innate Immune Responses. J Virol. 2022;96:e0151322. 10.1128/jvi.01513-22 PMC9683021 36314820

[41] Yu W, Liang J, Fang T, Jiang J, Zhao R, Li R, et al. EIF4A3 Acts on the PI3K-AKT-ERK1/2-P70S6K Pathway through FLOT1 to Influence the Development of Lung Adenocarcinoma. Mol Cancer Res. 2023;21:713–25. 10.1158/1541-7786.MCR-22-0984 PMC10320473 37011005

[42] Cordin O, Tanner NK, Doère M, Linder P, Banroques J. The newly discovered Q motif of DEAD-box RNA helicases regulates RNA-binding and helicase activity. Embo j. 2004;23:2478–87. 10.1038/sj.emboj.7600272 PMC449782 15201868

[43] Wang Y, Lu K, Xu Y, Xu S, Chu H, Fang X. Antibody-drug conjugates as immuno-oncology agents in colorectal cancer: targets, payloads, and therapeutic synergies. Front Immunol. 2025;16:1678907. 10.3389/fimmu.2025.1678907 PMC12620403 41256852

[44] Wang P, Xu S, Guo Q, Zhao Y. Discovery of PAK2 as a Key Regulator of Cancer Stem Cell in Head and Neck Squamous Cell Carcinoma Using Multi-Omic Techniques. Stem Cells Int. 2025;2025:1325262. 10.1155/sci/1325262 PMC12657082 41311809

[45] Ma J, Chen T, Wu S, Yang C, Bai M, Shu K, et al. iProX: an integrated proteome resource. Nucleic Acids Res. 2019;47:D1211–d7. 10.1093/nar/gky869 PMC6323926 30252093

[46] Chen T, Ma J, Liu Y, Chen Z, Xiao N, Lu Y, et al. iProX in 2021: connecting proteomics data sharing with big data. Nucleic Acids Res. 2022;50:D1522–d7. 10.1093/nar/gkab1081 PMC8728291 34871441

