# Supplementary material for: Targeting FEN1 disrupts EIF4A3-mediated MUC1-C autoregulation: a novel strategy to suppress lung adenocarcinoma progression
Source: Cell Death Dis. 2026 Sep 5;17(1):889. doi: 10.1038/s41419-026-09260-5 (PMC13675245; doi:10.1038/s41419-026-09260-5)
Supplement: Supplementary file 1 [file 41419_2026_9260_MOESM1_ESM.docx]

**Supplemental Information**

**Title:** Targeting FEN1 disrupts EIF4A3-mediated MUC1-C autoregulation: a novel strategy to suppress lung adenocarcinoma progression

**Authors:** Min Wu^1,2†*^, Benmeng Wu^1,2†^, Xuebing Yan^1,2†^, Zirui Wang^1,2^, Chunyan Mei^1,2^, Xiaoshan Huang^5^, Guorui Li^6^, Ji Pang^7^, Lin Yu^1,4*^, Jingjing Liu^1,3*^

**Contents**

**Supplemental Figures 1-10**

**Supplemental Table 1**

**Supplemental Figure 1**

**Figure S1.** FEN1 is upregulated in LUAD tissues and indicates poor prognosis. **A** FEN1 expression levels in LUAD grouped by different T stages. **B** FEN1 expression levels in LUAD grouped by different M stages. **C** FEN1 expression levels in LUAD grouped by different N stages. **D-H** The correlation analysis between FEN1 expression and clinical characteristics in LUAD patients. **I** Forest plots of the univariate and multivariate Cox regression analysis for predicting DFS in LUAD patients. **J** Nomogram for predicting 1-, 3-, and 5-year DSS in patients with LUAD (upper), and its corresponding calibration plots (lower). **P* < 0.05, ***P* < 0.01, ****P*< 0.001, ns, not significant.

**Supplemental Figure 2**

**Figure S2.** FEN1 downregulation induces senescence and impairs migration/invasion of LUAD cells. **A** Western blot analysis was performed to verify the knockdown efficiency of FEN1 in stable cell lines. **B** MTT assay was used to evaluate the effect of FEN1 downregulation on cell proliferation. **C** Flow cytometry was employed to detect the impact of FEN1 downregulation on cell cycle. **D** SA-β-gal staining was conducted to assess the effect of FEN1 downregulation on cell senescence. **E** Flow cytometry was utilized to determine the influence of FEN1 downregulation on cell apoptosis. **F-G** Transwell assay was carried out to investigate the effect of FEN1 downregulation on cell migration and invasion. Data are presented as means ± SD of three independent biological experiments. **P* < 0.05, ***P*< 0.01, ****P* < 0.001, *****P* < 0.0001, ns, not significant.

**Supplemental Figure 3**

**Figure S3.**  FEN1 and the PI3K/AKT pathway positively regulate MUC1-C expression in LUAD cells. **A** Western blot analysis of FEN1 and MUC1-C protein levels in wild-type LUAD cells (A549 and PC9). Cells were transfected with increasing amounts of a FEN1 overexpression plasmid (OE-FEN1: 0, 1, 2, and 4 μg, balanced with an empty vector) to demonstrate dose-dependent regulation. **B** Western blot analysis evaluating the “AKT → MUC1-C” feedback arm. Wild-type LUAD cells were treated with the AKT inhibitor MK-2206 (5 μM for A549 and 1 μM for PC9). Protein levels of p-AKT, total AKT, and MUC1-C were assessed. **C** Western blot analysis of wild-type LUAD cells transfected with a constitutively active AKT plasmid (ca-AKT) or control. Protein levels of p-AKT, total AKT, and MUC1-C were evaluated to confirm positive regulation by AKT activity. GAPDH was used as a loading control.

**Supplemental Figure 4**

**Figure S4.** FEN1 downregulation inhibits LUAD cells growth *in vivo*. **A** Tumor photographs of the sh-NC group and sh-FEN1#2 group. **B** Tumor volumes of the sh-NC group and sh-FEN1#2 group. **C** Tumor weights of the sh-NC group and sh-FEN1#2 group. **D** Representative images and quantitative results of cell senescence SA-β-gal staining in tumor tissues. **E** Representative images and quantitative results of FEN1, MUC1-C, and Ki67 IHC staining in tumor tissues. Data are shown as means ± SD. n=5, ***P* < 0.01, ****P* < 0.001, *****P* < 0.0001.

**Supplemental Figure 5**

**Figure S5.** **A** Flow cytometry analyzing the proportion of CD8+ T cells after co-culture of T cells with LUAD cells. **B-C** Flow cytometry detecting the intracellular expression of TNF-α and IFN-γ in CD4^+^ T cells after co-culture of T cells with LUAD cells. Data are presented as means ± SD of three independent biological experiments. ***P*< 0.01, ns, not significant.

**Supplemental Figure 6**

**Figure S6.** **A** Flow cytometry analyzing the proportion of CD8^+^ T cells after co-culture of T cells with FEN1-downregulated LUAD cells with ectopic MUC1-C overexpression. **B** Representative fluorescence images and quantitative analysis of Calcein-AM-labeled T cells recruited by conditioned media from the indicated A549 and PC9 cell groups in a Transwell chemotaxis assay (4 h incubation, 5 μm pore size). **C** Representative flow cytometry plots and quantitative analysis showing the percentage of PD-1^+^/ TIGIT^+^ cells specifically gated within the CD8^+^ T cell population following co-culture with A549 cells under the indicated conditions. Data are presented as means ± SD of three independent biological experiments. ***P*< 0.01, ****P* < 0.001, *****P* < 0.0001, ns, not significant.

**Supplemental Figure 7**

**Figure S7.** **A** Flow cytometry determining the intracellular expression of TNF-α and IFN-γ in intratumoral CD4^+^ T cells. Data are shown as means ± SD. n=4, **P*< 0.05, ns, not significant.

**Supplemental Figure 8**

**Figure S8. A** Endogenous Co-IP assay showing the interaction between FEN1 and EIF4A3 in 293T cell lysates treated with or without Benzonase nuclease. Total cell lysates were pre-treated with Benzonase to digest DNA and RNA prior to immunoprecipitation. n=3. **B** Western blot analysis of the input lysates from nuclear and cytoplasmic fractions in Control and FEN1-KD cells. Lamin A/C and GAPDH serve as nuclear and cytoplasmic markers, respectively. n=3.**C** Endogenous Co-IP assays of nuclear and cytoplasmic fractions from PC9 cells using anti-FEN1 and anti-EIF4A3 antibodies. n=3.**D** Immunofluorescence staining demonstrating the colocalization of FEN1 and EIF4A3 in human LUAD tissues. n=10.

**Supplemental Figure 9**

**Figure S9. A-B** Pearson correlation analysis between FEN1 and PD-L1 mRNA expression in the TCGA-LUAD cohort, stratified into EGFR wild-type (A) and EGFR mutant groups (B). **C** Kaplan-Meier survival curves for overall survival in patients stratified by FEN1 and MUC1-C co-expression. **D** Overall survival analysis based on FEN1, MUC1-C, and PD-L1 co-expression (the “triple-positive” signature, defined by mean expression).

**Supplemental Figure 10**

**Figure S10.** Direct pharmacological inhibition of MUC1-C by GO-203 inhibits cell migration and invasion, induces senescence, and attenuates PD-L1 in LUAD cells. **A** Cell viability assays of A549 and PC9 cells treated with varying concentrations of the specific MUC1-C peptide inhibitor GO-203 (0-20 μM) for 24 h. For subsequent experiments (B-E), LUAD cells were treated with the maximum non-toxic concentrations of GO-203 (2.5 μM for A549 and 10 μM for PC9) to exclude the interference of cytotoxicity. **B** Transwell migration assays demonstrating the inhibitory effect of GO-203 on LUAD cell migration. **C** Transwell invasion assays demonstrating the inhibitory effect of GO-203 on LUAD cell invasion. **D** SA-β-gal staining showing the induction of cellular senescence in A549 and PC9 cells following GO-203 treatment.
**E** Western blot analysis demonstrating the suppression of immune-related signaling, evidenced by the downregulation of PD-L1 protein expression in LUAD cells treated with GO-203. GAPDH was used as a loading control. Data are presented as means ± SD of at least three independent biological experiments. **P* < 0.05, ***P*< 0.01, ****P* < 0.001, ns, not significant.

**Supplemental Table 1	List of antibodies used in this study**

| **Antibody** | **Company** | **Catalog Number** |
| --- | --- | --- |
| GAPDH | Proteintech | 60004-1-Ig |
| FEN1 | Proteintech | 14768-1-AP |
| Phospho-Akt (Ser473) (D9E) XP® Rabbit mAb | Cell Signaling Technology | 4060T |
| Akt (pan) (11E7) Rabbit mAb | Cell Signaling Technology | 4685S |
| Phospho-PI3 Kinase p85 (Tyr458)/p55 (Tyr199) Antibody | Cell Signaling Technology | 4228T |
| PI3 Kinase p85 Alpha | Proteintech | 30092-1-AP |
| Mucin 1(VU4H5) | Santa Cruz Biotechnology | sc-7313 |
| MUC1/CA15-3 C-terminal Polyclonal antibody | Proteintech | 23614-1-AP |
| NF-κB p65 (D14E12) XP® Rabbit mAb | Cell Signaling Technology | 8242T |
| PD-L1 Recombinant Rabbit mAb | Starter Biotechnology | SDT-R017 |
| CD3 Monoclonal Antibody (UCHT1), PE, eBioscience™ | ThermoFisher | 12-0038-42 |
| FITC anti-human CD4 Antibody | Biolegend | 317408 |
| APC Mouse Anti-Human CD8 | BD Biosciences | 566852 |
| PE/Cyanine7 anti-human/mouse Granzyme B Recombinant | Biolegend | 372213 |
| Brilliant Violet 421™ anti-human TNF-α | Biolegend | 502931 |
| RB705 Mouse Anti-Human IFN-γ | BD Biosciences | 570248 |
| PD-L1 (D4H1Z) Rabbit mAb | Cell Signaling Technology | 60475S |
| FITC Rat Anti-Mouse CD45 | BD Biosciences | 553079 |
| BV510 Hamster Anti-Mouse CD3e | BD Biosciences | 563024 |
| APC Rat Anti-Mouse CD4 | BD Biosciences | 553051 |
| PerCP-Cy™5.5 Rat Anti-Mouse CD8a | BD Biosciences | 551162 |
| PE Rat Anti-Mouse TNF-α | BD Biosciences | 561063 |
| PE Rat Anti-Mouse IFN-γ | BD Biosciences | 562020 |
| eIF4AIII (B-2) | Santa Cruz Biotechnology | sc-365549 |
| Pdcd-4 (B-4) | Santa Cruz Biotechnology | sc-376430 |
| 6*His, His-tag Polyclonal antibody | Proteintech | 10001-0-AP |
| DYKDDDDK tag Monoclonal antibody (Binds to FLAG® tag epitope) | Proteintech | 66008-4-Ig |
| Ki67 | Absin | abs130135 |
| Anti-mouse IgG,  HRP-linked Antibody | Cell Signaling Technology | #7076 |
| Anti-rabbit IgG,  HRP-linked Antibody | Cell Signaling Technology | #7074 |
| CoraLite488-conjucated Goat Anti-Rabbit IgG(H+L) | Proteintech | SA00013-2 |
| CoraLite594-conjucated Goat Anti-Mouse IgG(H+L) | Proteintech | SA00013-3 |
| Anti-Human TIGIT Antibody | ZENBIO | 30338 |
| Anti-Human PD-1 Antibody | ZENBIO | 30036 |
| Lamin A/C | ZENBIO | R26947 |
| Puromycin Rabbit mAb | Abclonal | A23031 |
| RPL10a | Proteintech | 16681-1-AP |
