# Supplementary material for: Targeting FEN1 disrupts EIF4A3-mediated MUC1-C autoregulation: a novel strategy to suppress lung adenocarcinoma progression
Source: Cell Death Dis. 2026 Sep 5;17(1):889. doi: 10.1038/s41419-026-09260-5 (PMC13675245; doi:10.1038/s41419-026-09260-5)
Supplement: Supplementary file 2 [file 41419_2026_9260_MOESM2_ESM.docx]

**Figure S2 A**

A549

1	2	3

FEN1

GAPDH

PC9

1	2	3

FEN1

GAPDH

The original images for Figure S2 A

Lane1: sh-NC

Lane2: sh-FEN1#1

Lane3: sh-FEN1#2

**Figure 2 B**

A549

1	2	3

p-AKT

AKT

p-PI3K

PI3K

PDCD4

MUC1

MUC1-C

GAPDH

PC9

1	2	3

p-AKT

AKT

p-PI3K

PI3K

PDCD4

MUC1

MUC1-C

GAPDH

The original images for Figure 2 B

Lane1: sh-NC

Lane2: sh-FEN1#1

Lane3: sh-FEN1#2

**Figure 2 D**

A549

1	2	3

p-AKT

AKT

p-PI3K

PI3K

MUC1-C

FEN1

GAPDH

PC9

1	2	3

p-AKT

AKT

p-PI3K

PI3K

MUC1-C

FEN1

GAPDH

The original images for Figure 2 D

Lane1: sh-NC

Lane2: sh-FEN1#2

Lane3: sh-FEN1#2+oe-MUC1-C

**Figure S3A- left**

A549

1	2	3	4

FEN-1

MUC1-C

GAPDH

The original images for Figure S3A-left

Lane1: Control

Lane2: 1μg

Lane3: 2μg

Lane4: 4μg

**Figure S3A-right**

PC9

1	2	3	4

FEN-1

MUC-1

GAPDH

The original images for Figure S3A-right

Lane1: Control

Lane2: 1μg

Lane3: 2μg

Lane4: 4μg

**Figure S3B- left**

A549

1	2

p-AKT

AKT

MUC1-C

GAPDH

The original images for Figure S3B-left

Lane1: Control

Lane2: MK-2206

**Figure S3B-right**

PC9

1	2

p-AKT

AKT

MUC1-C

GAPDH

The original images for Figure S3B-right

Lane1: Control

Lane2: MK-2206

**Figure S3C-left**

A549

1	2

p-AKT

AKT

MUC1-C

GAPDH

The original images for Figure S3C-left

Lane1: Control

Lane2: ca-AKT

**Figure S3C-right**

PC9

1	2

p-AKT

AKT

MUC1-C

GAPDH

The original images for Figure S3C-right

Lane1: Control

Lane2: ca-AKT

**Figure 3 A**

A549

1	2	3

p65

PD-L1

MUC1-C

GAPDH

PC9

1	2	3

p65

PD-L1

MUC1-C

GAPDH

The original images for Figure 3 A

Lane1: sh-NC

Lane2: sh-FEN1#1

Lane3: sh-FEN1#2

**Figure 4 A**

A549

1	2	3

p65

PD-L1

MUC1-C

GAPDH

PC9

1 2 3

p65

PD-L1

MUC1-C

GAPDH

The original images for Figure 4A

Lane1: sh-NC

Lane2: sh-FEN1#2

Lane3: sh-FEN1#2+oe-MUC1-C

**Figure 5 A**

LLC

1	2	3

FEN1

MUC1-C

PD-L1

GAPDH

The original images for Figure 5A

Lane1: sh-NC

Lane2: sh-FEN1#1

Lane3: sh-FEN1#2

**Figure 6 B**

HEK293T (up)

1	2	3

IB: His

IB: Flag

The original images for Figure 6 B-HEK293T

Lane1: Input

Lane2: IgG

Lane3: IP: Flag

HEK293T (down)

1	2	3

IB: Flag

IB: His

The original images for Figure 6 B-HEK293T

Lane1: Input

Lane2: IgG

Lane3: IP: Flag

A549 (up)

1	2	3

IB: EIF4A3

IB: FEN1

The original images for Figure 6 B-A549

Lane1: Input

Lane2: IgG

Lane3: IP: FEN1

A549 (down)

1	2	3

IB: FEN1

IB: EIF4A3

The original images for Figure 6 B-A549

Lane1: Input

Lane2: IgG

Lane3: IP: EIF4A3

PC9 (up)

1	2	3

IB: EIF4A3

IB: FEN1

The original images for Figure 6 B-PC9

Lane1: Input

Lane2: IgG

Lane3: IP: FEN1

PC9 (down)

1	2	3

IB: FEN1

IB: EIF4A3

The original images for Figure 6 B-PC9

Lane1: Input

Lane2: IgG

Lane3: IP: EIF4A3

**Figure 6 C**

A549

1	2	3

EIF4A3

GAPDH

PC9

1	2	3

EIF4A3

GAPDH

The original images for Figure 6 C

Lane1: sh-NC

Lane2: sh-FEN1#1

Lane3: sh-FEN1#2

**Figure 6 D-left**

1 2	3 4	5 6

IB: FEN1

IB: EIF4A3

IB: EIF4A3

IB: FEN1

The original images for Figure 6 D-left

Lane1: sh-NC-Input

Lane2: sh-FEN1#2-Input

Lane3: sh-NC-IgG

Lane4: sh-FEN1#2-IgG

Lane5: sh-NC-IP

Lane6: sh-FEN1#2-IP

**Figure 6 D-right**

1 2 3 4 5 6

IB: FEN1

IB: EIF4A3

IB: EIF4A3

IB: FEN1

The original images for Figure 6 D-right

Lane1: sh-NC-Input

Lane2: sh-FEN1#2-Input

Lane3: sh-NC-IgG

Lane4: sh-FEN1#2-IgG

Lane5: sh-NC-IP

Lane6: sh-FEN1#2-IP

**Figure 6 G**

A549	PC9

1	2	3	4	5	6

EIF4A3

FEN1

GAPDH

GAPDH

The original images for Figure 6 G

Lane1: si-NC

Lane2: si-FEN1

Lane3: si-FEN1+OE-EIF4A3

Lane4: si-NC

Lane5: si-FEN1

Lane6: si-FEN1+OE-EIF4A3

**Figure 6 H**

A549	PC9

1	2	3	4	5	6

Puromycin

The original images for Figure 6H

Lane1: si-NC

Lane2: si-FEN1

Lane3: si-FEN1+OE-EIF4A3

Lane4: si-NC

Lane5: si-FEN1

Lane6: si-FEN1+OE-EIF4A3

**Figure 7 B-left**

HEK293T (whole cell lysates)

1	2	3

IB: His

IB: Flag

HEK293T (IP: Flag-FEN1)

1	2	3

IB: His

IB: Flag

The original images for Figure 7B-left

Lane1: transfect Flag-FEN1+ His-EIF4A3 full length

Lane2: transfect Flag-FEN1+ His-EIF4A3 N-terminal

Lane3: transfect Flag-FEN1+ His-EIF4A3 C-terminal

**Figure 7 B-right**

HEK293T (whole cell lysates)

1	2	3

IB: His

IB: Flag

HEK293T (IP: His-EIF4A3)

1	2	3

IB: His

IB: Flag

The original images for Figure 7B-right

Lane1: transfect His-EIF4A3+Flag-FEN1 full length

Lane2: transfect His-EIF4A3+Flag-FEN1 N-terminal

Lane3: transfect His-EIF4A3+Flag-FEN1 C-terminal

**Figure 7 C-left**

A549

1	2	3

MUC1-C

GAPDH

PC9

1	2	3

MUC1-C

GAPDH

The original images for Figure 7C-left

Lane1: transfect Flag-FEN1+ His-EIF4A3 full length

Lane2: transfect Flag-FEN1+ His-EIF4A3 N-terminal

Lane3: transfect Flag-FEN1+ His-EIF4A3 C-terminal

**Figure 7 C-right**

A549

1	2	3

MUC1-C

GAPDH

PC9

1	2	3

MUC1-C

GAPDH

The original images for Figure 7C-right

Lane1: transfect His-EIF4A3+Flag-FEN1 full length

Lane2: transfect His-EIF4A3+Flag-FEN1 N-terminal

Lane3: transfect His-EIF4A3+Flag-FEN1 C-terminal

**Figure 7 D**

1 2 3 4	5 6

IB: Flag

IB: His

IB: His

IB: Flag

The original images for Figure 7 D

Lane1: WT-Input

Lane2: MUT-Input

Lane3: WT -IgG

Lane4: MUT -IgG

Lane5: WT -IP

Lane6: MUT -IP

**Figure 7 E**

A549

1	2	3

MUC1-C

GAPDH

PC9

1	2	3

MUC1-C

GAPDH

The original images for Figure 7E

Lane1: si-FEN1

Lane2: si-FEN1+WT

Lane3: si-FEN1+MUT

**Figure 7 J**

A549	PC9

1	2	3	4	5	6

MUC1-C

GAPDH

The original images for Figure 7J

Lane1: sh-NC

Lane2: sh-FEN1#2

Lane3: sh-FEN1#2+OE-EIF4A3

Lane4: sh-NC

Lane5: sh-FEN1#2

Lane6: sh-FEN1#2+OE-EIF4A3

**Figure S8 A**

1 2	3 4	5 6

IB: FEN1

IB: EIF4A3

IB: EIF4A3

IB: FEN1

The original images for Figure S8A

Lane1: Con-Input

Lane2: Benzonase-Input

Lane3: Con-IgG

Lane4: Benzonase-IgG

Lane5: Con-IP

Lane6: Benzonase-IP

**Figure S8 B**

A549

1 2	3 4

**1**

Lamin A/C

GAPDH

PC9

1 2	3 4

Lamin A/C

GAPDH

The original images for Figure S8B

Lane1: sh-NC-Nucleus

Lane2: sh-FEN1#2-Nucleus

Lane3: sh-NC-Cytoplasm

Lane4: sh-FEN1#2-Cytoplasm

**Figure S8 C-left**

1 2	3	4	5	6

IB:FEN1

IB: EIF4A3

IB: EIF4A3

IB: FEN1

The original images for Figure S8C-left

Lane1: sh-NC-Input

Lane2: sh-FEN1#2-Input

Lane3: sh-NC-IgG

Lane4: sh-FEN1#2-IgG

Lane5: sh-NC-IP

Lane6: sh-FEN1#2-IP

**Figure S8C-right**

1 2	3	4	5	6

IB: FEN1

IB: EIF4A3

IB: EIF4A3

IB: FEN1

The original images for Figure S8C-right

Lane1: sh-NC-Input

Lane2: sh-FEN1#2-Input

Lane3: sh-NC-IgG

Lane4: sh-FEN1#2-IgG

Lane5: sh-NC-IP

Lane6: sh-FEN1#2-IP

**Figure S10 E- left**

A549

1	2

PD-L1

GAPDH

The original images for Figure S10E-left

Lane1: Control

Lane2: GO-203

**Figure S10 E- right**

PC9

1	2

PD-L1

GAPDH

The original images for Figure S10E-right

Lane1: Control

Lane2: GO-203
